# Appendicular-to-trunk lean mass ratio resolves the height-based normalization paradox in low muscle mass and metabolic syndrome: A population-based cross-sectional study

**DOI:** 10.1371/journal.pone.0357017

**Published:** 2026-08-25

**Authors:** Jung Ah Lim, Sung Hoon Yu, Jung Hwan Park, Shinje Moon, Chang Beom Lee, Sangmo Hong

**Affiliations:** Department of Internal Medicine, Hanyang University College of Medicine, Seoul, Republic of Korea; The University of Alabama at Birmingham Heersink School of Medicine, UNITED STATES OF AMERICA

## Abstract

Whether height-based normalization indices for appendicular lean mass (ALM) are metabolically neutral remains uncertain, because such indices may be influenced by adiposity. We evaluated ALM normalized to trunk lean mass (ALM/TLM) — both with bone mineral content excluded — as a normalization strategy potentially less susceptible to adiposity-related distortion. Using the Korea National Health and Nutrition Examination Survey 2008–2010, we analyzed 3,176 adults aged ≥65 years (1,373 men; 1,803 women) for body composition by dual-energy X-ray absorptiometry. Sex-specific Class I and Class II cut-offs were derived from a healthy young reference group aged 20–39 years (n = 3,727) at 1 SD and 2 SD below the young-adult mean. Metabolic syndrome (MetS) was defined by NCEP-ATP III criteria with population-specific waist cut-offs. Survey-weighted regression models were adjusted for age, smoking, alcohol, and physical activity. The appendicular skeletal muscle mass index (ASMI; ALM/height^2^) correlated positively with fat mass index (FMI) (men r=+0.288; women r=+0.358; both p < 0.001), whereas ALM/TLM correlated negatively with FMI (men r=−0.199; women r=−0.243; both p < 0.001). ASMI Class I low muscle mass showed an inverse association with MetS (men odds ratio [OR] 0.496, 95% confidence interval [CI] 0.388–0.636; women OR 0.475, 95% CI 0.328–0.686). In contrast, ALM/TLM Class II showed positive associations with MetS (men OR 2.779, 95% CI 2.074–3.722; women OR 2.701, 95% CI 1.680–4.340; both p < 0.001). The inverse ALM/TLM–HOMA-IR association persisted after FMI adjustment (men β=−0.131, p < 0.001; women β=−0.065, p = 0.007). ASMI correlated positively with adiposity, while ASMI-defined low muscle mass was inversely associated with MetS, suggesting that ASMI may partly reflect body size/adiposity rather than relative muscle depletion alone; this inverse association should not be interpreted as protective. By contrast, ALM/TLM-defined low muscle mass showed consistently positive associations with MetS across adiposity strata, supporting further evaluation of ALM/TLM, although its clinical validity remains unestablished.

## Introduction

Skeletal muscle is the principal site of insulin-stimulated glucose disposal [1], making muscle depletion biologically plausible as a determinant of insulin resistance and metabolic syndrome (MetS). However, whether reduced skeletal muscle mass contributes to metabolic risk independently of adiposity remains an important but unresolved question [1,2]. This question is difficult to address directly because commonly used lean-mass indices are not independent of adiposity. Greater abdominal adiposity may coexist with higher ALM but poorer muscle quality [3], and fat mass and fat-free mass tend to increase concurrently and should be interpreted in relation to one another [4]. Consequently, the apparent metabolic significance of low muscle mass may differ substantially depending on how ALM is normalized, making the muscle-specific contribution to metabolic risk difficult to isolate when the index used remains dependent on adiposity.

Among available indices, the appendicular skeletal muscle mass index (ASMI; ALM/height^2^) is the most widely used, but height does not reflect body composition. Individuals with greater adiposity often have greater absolute lean mass, which may keep ASMI above its cut-off despite relative appendicular muscle depletion. Conversely, individuals with lower adiposity may fall below the cut-off despite having lower metabolic risk [5,6]. As a result, ASMI may yield paradoxical inverse associations with metabolic risk in populations with a high prevalence of adiposity. Alternative indices such as ALM/body mass index (BMI) and ALM/weight produce more metabolically coherent associations by incorporating body mass into the denominator [5–7]. However, because adiposity is embedded directly in the denominator, these indices reflect a combination of muscle depletion and adiposity rather than muscle depletion assessed independently of fat mass [8]. These considerations suggest that the choice of normalization strategy may have a substantial effect on the apparent relationship between muscle mass and metabolic risk.

We therefore explored ALM normalized to trunk lean mass (TLM) as an alternative denominator strategy. Rather than relying on height alone or directly incorporating body weight or BMI, TLM is used as an internal reference in ALM/TLM and may be less directly influenced by adiposity than conventional height-based normalization. Given that dual-energy X-ray absorptiometry (DXA)-derived trunk lean mass is largely composed of visceral organs rather than peripheral skeletal muscle [9,10], its denominator may respond differently to fat mass accumulation than height-based denominators. We hypothesized that ALM/TLM would present a more metabolically coherent association pattern than ASMI in older adults. Accordingly, in a nationally representative sample of adults aged ≥65 years, we examined the associations of ALM/TLM and ASMI with MetS and insulin resistance. We also compared the index behavior of ALM/TLM, ASMI, ALM/BMI, and ALM/weight, and assessed whether any observed association for ALM/TLM persisted after adjustment for measured fat mass index.

## Materials and methods

### Ethics statement

This secondary analysis used publicly available, anonymized data from the Korea National Health and Nutrition Examination Survey (KNHANES) 2008–2010. The original KNHANES 2008–2010 protocol was reviewed and approved by the Institutional Review Board of the Korea Centers for Disease Control and Prevention (approval numbers 2008-04EXP-01-C, 2009-01CON-03-2C, and 2010-02CON-21-C), and written informed consent was obtained from all participants at the time of each respective survey. Because the present analysis used only publicly available, anonymized data, separate ethics approval and additional informed consent were not required for this secondary analysis. The study was conducted in accordance with the Declaration of Helsinki and is reported in accordance with the STROBE guideline (S1 Checklist).

### Study design and participants

This cross-sectional study used data from KNHANES 2008–2010. KNHANES uses a clustered, multistage, stratified, probability sampling design with annual rolling samples [11]. The primary analysis cohort comprised 3,176 adults aged ≥65 years (1,373 men; 1,803 women). Participants were excluded for missing DXA or laboratory data, or a history of liver, renal, or pulmonary disease, or malignancy. Participants with metabolic diseases, including those meeting MetS criteria, were retained, as metabolic outcomes constituted the primary endpoints. Medication use affecting muscle metabolism (e.g., corticosteroids, anabolic agents, and hormonal therapies) was not systematically recorded in the KNHANES 2008–2010 dataset, so it was not possible to control for this variable in the analysis.

A separate young reference group for deriving cut-off values comprised adults aged 20–39 years. In addition to the exclusion criteria above, individuals with metabolic disease were excluded from this group. Metabolic disease was defined as self-reported physician-diagnosed diabetes, hypertension, dyslipidemia, or MetS, based on the corresponding KNHANES variables. After these exclusions, 3,727 participants (1,768 men; 1,959 women) were included in the young reference group.

### DXA measurements and body composition

Whole-body DXA was performed using a QDR 4500A fan-beam densitometer (Hologic, Inc., Bedford, MA, USA), operated in accordance with the manufacturer’s procedures and analyzed using Hologic Discovery software (version 13.1) in its default configuration. ALM was defined as the sum of arm and leg lean soft tissue mass; TLM was defined as trunk lean soft tissue mass. ALM and TLM were derived from DXA-reported lean soft tissue mass with bone mineral content excluded from each compartment. ALM/TLM was calculated as ALM divided by TLM. Fat mass index (FMI) is equivalent to total fat mass (kg)/height^2^ (m^2^).

### Low muscle mass definitions

Sex-specific cut-offs were derived from the young reference group using thresholds at 1 SD and 2 SD (Classes I and II) below the sex-specific young-adult mean. For ALM/TLM, the cut-offs were <0.877 and <0.812 in men and <0.756 and <0.693 in women for Classes I and II, respectively. For ASMI, the corresponding cut-offs were <7.031 and <6.155 kg/m^2^in men and <5.006 and <4.266 kg/m^2^in women. Full derivation is provided in S1 and S2 Tables (S1 File).

### Metabolic syndrome and insulin resistance

MetS was defined according to the National Cholesterol Education Program Adult Treatment Panel III (NCEP-ATP III) criteria [12], with population-specific waist circumference thresholds (men ≥ 90 cm; women ≥85 cm) [13]. Participants were classified as having MetS if they met three or more of the following: abdominal obesity, triglycerides ≥150 mg/dL, HDL cholesterol <40 mg/dL (men) or <50 mg/dL (women), blood pressure ≥130/85 mmHg, or fasting plasma glucose (FPG) ≥100 mg/dL. Insulin resistance was assessed using the homeostasis model assessment of insulin resistance (HOMA-IR) = [fasting insulin (μU/mL) × fasting plasma glucose (mg/dL)]/405 [14]. FMI categories were defined using thresholds approximated to match established BMI category prevalences in the source population, based on DXA-derived body composition reference data [15,16]: fat deficit (<3 kg/m^2^ in men, < 5 kg/m^2^in women), normal (3–6/5–9 kg/m^2^), fat excess (6–9/9–13 kg/m^2^), and obesity (>9/ > 13 kg/m^2^

### Statistical analysis

All statistical analyses were performed using IBM SPSS Statistics version 29.0 (IBM Corp., Armonk, NY, USA). Continuous variables are presented as mean ± SD and categorical variables as proportions. Between-group comparisons used the independent-samples t-test and chi-square test; these analyses were unweighted to maintain consistency with reported p-values (Table 1), and survey-weighted baseline estimates are in S3 Table (S1 File). Pearson correlations, unweighted linear regression (standardized β; Table 3), and the trunk-to-leg lean mass ratio comparison (S8 Table in S1 File) were performed without survey weighting to characterize index behavior. HOMA-IR was log-transformed due to right skew. For primary inferential analyses, sampling weights (wt_tot), stratification variables (kstrata), and primary sampling units (psu) were incorporated using the KNHANES analytic guidelines. Logistic regression for MetS (Tables 4 and 5; S4–S6 Tables in S1 File) used the Complex Samples procedure (CSLOGISTIC) with cluster-robust standard errors. The simultaneous model (Table 5) included FMI category and ALM/TLM class as concurrent predictors. Subgroup analyses were stratified by BMI and FMI category with linear trend tests. Physical activity-stratified analyses used a binary classification (sedentary vs. any regular physical activity, S6 Table in S1 File), and each index was additionally modeled using 1-SD change with and without FMI adjustment (S5 Table in S1 File). Within-sex low muscle mass prevalence by FMI category is provided in S7 Table (S1 File). In a supplementary analysis, participants were cross-classified into four mutually exclusive categories based on current smoking and alcohol consumption ≥2 times/week; this joint variable was used both as an adjustment covariate and, where estimable, to assess effect modification of the ALM/TLM–MetS association (sex-stratified complex-samples logistic regression; S9 Table in S1 File). In a complete-case dietary subsample, we examined whether additional adjustment for the percentage of energy derived from fat changed the ALM/TLM–MetS estimates (S10 Table in S1 File). Two-sided p < 0.05 was considered statistically significant.

## Results

### Baseline characteristics

Baseline characteristics are presented in Table 1 (unweighted); survey-weighted estimates are provided in S3 Table (S1 File). Ages did not differ significantly between men and women (71.7 ± 5.0 vs. 71.9 ± 5.1 years; p = 0.488). Men had greater ALM (19.4 ± 2.7 vs. 13.5 ± 1.9 kg) and TLM (22.6 ± 3.0 vs. 17.4 ± 2.4 kg), while women had higher FMI (8.2 ± 2.3 vs. 5.1 ± 1.7 kg/m^2^and body fat (34.0 ± 5.9 vs. 22.5 ± 5.4%). Fasting plasma glucose was slightly higher in men (105.3 ± 28.9 vs. 103.0 ± 24.6 mg/dL; p = 0.018). Survey-weighted MetS prevalence was 42.5% in men and 57.1% in women (p < 0.001); the corresponding unweighted values in Table 1 were 40.6% and 53.5%, which differed significantly by sex (p < 0.001, chi-square).

**Table 1:** Baseline characteristics of study participants aged ≥65 years by sex (KNHANES 2008–2010; n = 3,176).

Variable	Men (n = 1,373)	Women (n = 1,803)	p value
**Demographics**			
Age (years)	71.7 ± 5.0	71.9 ± 5.1	0.488
65–69 years (%)	39.3	39.5	
70–74 years (%)	27.3	25.9	
75–79 years (%)	25.5	25.5	
≥80 years (%)	8.0	9.1	
**Anthropometrics**			
Height (cm)	164.9 ± 5.8	150.9 ± 5.8	<0.001
Weight (kg)	62.8 ± 9.4	55.0 ± 9.1	<0.001
BMI (kg/m²)	23.1 ± 2.9	24.1 ± 3.4	<0.001
Waist circumference (cm)	84.5 ± 9.0	83.2 ± 9.8	<0.001
**Lifestyle factors**			
Current smoker (%)	25.7	5.0	<0.001
Alcohol ≥2 × /week (%)	37.3	5.4	<0.001
Regular physical activity (%)	14.0	13.2	0.557
**Body composition – DXA**			
ALM (kg)	19.4 ± 2.7	13.5 ± 1.9	<0.001
TLM (kg)	22.6 ± 3.0	17.4 ± 2.4	<0.001
ALM/height² – ASMI (kg/m²)	7.16 ± 0.80	5.91 ± 0.66	<0.001
ALM/TLM	0.86 ± 0.07	0.78 ± 0.07	<0.001
FMI (kg/m²)	5.1 ± 1.7	8.2 ± 2.3	<0.001
Body fat (%)	22.5 ± 5.4	34.0 ± 5.9	<0.001
**Metabolic parameters**			
Systolic BP (mmHg)	130.1 ± 17.6	132.6 ± 17.8	<0.001
Diastolic BP (mmHg)	76.9 ± 9.9	77.2 ± 9.8	0.528
Fasting plasma glucose (mg/dL)	105.3 ± 28.9	103.0 ± 24.6	0.018
Total cholesterol (mg/dL)	180.2 ± 34.6	199.5 ± 36.4	<0.001
HDL cholesterol (mg/dL)	48.4 ± 12.8	50.0 ± 11.6	<0.001
Triglycerides (mg/dL)	141.0 ± 95.4	146.8 ± 83.7	0.071
HOMA-IR	2.5 ± 1.9	2.8 ± 3.3	<0.001
Metabolic syndrome (%)	40.6	53.5	<0.001
**Low muscle mass prevalence**			
ASMI-defined, Class I (cumul. %)	43.9	8.0	<0.001
ASMI-defined, Class II (%)	9.7	0.4	<0.001
ALM/TLM-defined, Class I (cumul. %)	57.7	40.2	<0.001
ALM/TLM-defined, Class II (%)	21.1	11.1	<0.001

*Values are mean ± SD or unweighted prevalence (%). p-values from independent-samples t-test (continuous) or chi-square test (categorical). Survey-weighted estimates are provided in S3 Table (* *S1 File* *). Class I low muscle mass: cumulative (includes Class II).*

*ASMI = appendicular skeletal muscle mass index (ALM/height* ^2^ *, kg/m* ^2^ *); ALM/TLM = appendicular lean mass to trunk lean mass ratio; FMI = fat mass index (kg/m* ^2^ *); HOMA-IR = homeostasis model assessment of insulin resistance; HDL = high-density lipoprotein; BMI = body mass index; DXA = dual-energy X-ray absorptiometry; SD = standard deviation.*

### Prevalence of low muscle mass

**Fig 1:**
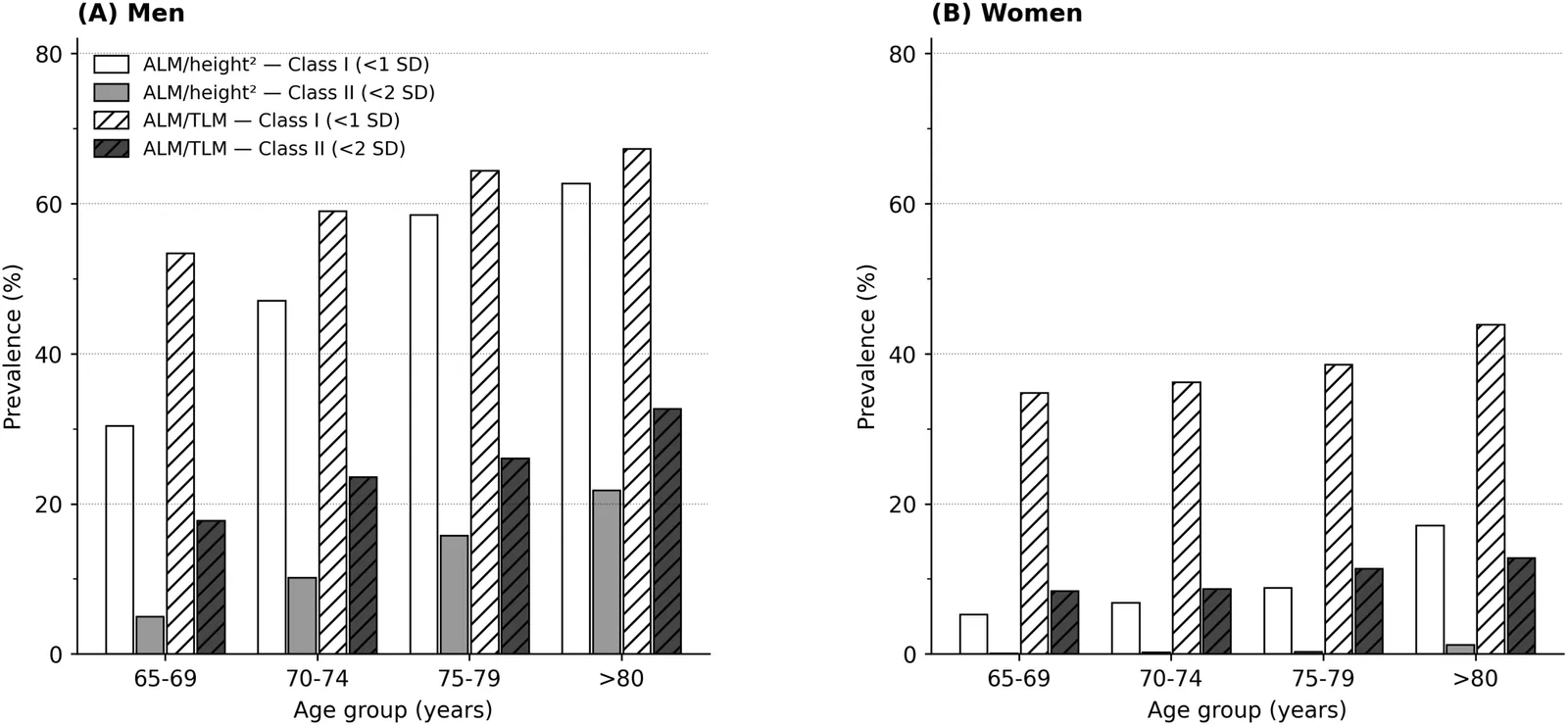
Survey-weighted prevalence of low muscle mass by age group in adults aged ≥65 years. **(a)** Men. **(b)** Women. KNHANES 2008–2010 (n = 3,176). ASMI = appendicular skeletal muscle mass index (ALM/height^2^kg/m^2^); ALM/TLM = appendicular lean mass to trunk lean mass ratio. Class I and Class II thresholds correspond to 1 SD and 2 SD below the sex-specific young-adult reference mean (n = 3,727; aged 20–39 years). Class I prevalence is cumulative (includes Class II).

The prevalence of low muscle mass, stratified into four age groups (65–69, 70–74, 75–79, and ≥80 years), is shown in Fig 1. ASMI Class I low muscle mass (cumulative, below 1 SD) was 43.9% in men and 8.0% in women; Class II was 9.7% and 0.4%, respectively. ALM/TLM Class I low muscle mass (cumulative, below 1 SD) was 57.7% in men and 40.2% in women; Class II was 21.1% and 11.1%, respectively. Both indices showed age-dependent increases across all four strata in both sexes. The sex disparity in ASMI-based prevalence was substantially attenuated with ALM/TLM.

### Associations between body composition indices, adiposity, and metabolic variables

Pearson correlations are presented in Table 2. ASMI was positively correlated with FMI (men r=+0.288; women r=+0.358; both p < 0.001). By contrast, ALM/TLM was negatively correlated with FMI (men r=−0.199; women r=−0.243; both p < 0.001). ALM/TLM showed negative correlations with all adverse metabolic parameters; ASMI showed positive correlations with diastolic BP and HOMA-IR.

**Table 2:** Pearson correlations between body composition indices (ASMI and ALM/TLM), FMI, and metabolic/anthropometric parameters.

	Men			Women		
	**ASMI**	**ALM/TLM**	**FMI**	**ASMI**	**ALM/TLM**	**FMI**
ALM/height²	1.000	+0.406**	+0.288**	1.000	+0.367**	+0.358**
ALM/TLM	+0.406**	1.000	−0.199**	+0.367**	1.000	−0.243**
FMI	+0.288**	−0.199**	1.000	+0.358**	−0.243**	1.000
Age	−0.293**	−0.124**	−0.019	−0.158**	−0.064*	−0.122**
BMI	+0.714**	−0.103**	+0.839**	+0.653**	−0.195**	+0.918**
Waist circ.	+0.553**	−0.146**	+0.800**	+0.507**	−0.199**	+0.823**
%Fat	+0.016	−0.224**	+0.943**	+0.042	−0.249**	+0.919**
Systolic BP	+0.038	−0.078*	+0.132**	+0.041	−0.092**	+0.083**
Diastolic BP	+0.102**	−0.009	+0.133**	+0.082**	−0.099**	+0.133**
HDL cholesterol	−0.124**	+0.147**	−0.302**	−0.095**	+0.055*	−0.110**
Triglycerides	+0.078*	−0.114**	+0.237**	+0.027	−0.145**	+0.114**
FPG	−0.017	−0.210**	+0.133**	+0.076*	−0.123**	+0.150**
HOMA-IR	+0.099**	−0.199**	+0.365**	+0.076*	−0.112**	+0.201**

Unweighted Pearson correlations. *p < 0.05; **p < 0.001.

*ASMI = appendicular skeletal muscle mass index (ALM/height* ^2^ *, kg/m* ^2^ *); ALM/TLM = appendicular lean mass to trunk lean mass ratio; FMI = fat mass index (kg/m* ^2^ *); FPG = fasting plasma glucose; HOMA-IR = homeostasis model assessment of insulin resistance; HDL = high-density lipoprotein; BP = blood pressure; BMI = body mass index.*

Multiple linear regression (Table 3) showed ASMI was positively associated with FMI (men β=+0.304, R^2^ = 0.112; women β=+0.348, R^2^= 0.141; both p < 0.001), while ALM/TLM was negatively associated (men β=−0.205, R^2^ = 0.069; women β=−0.255, R^2^= 0.089; both p < 0.001). ASMI was positively associated with HOMA-IR (men β=+0.104, R^2^= 0.021; women β=+0.080, R^2^= 0.008; both p < 0.001), while ALM/TLM was negatively associated (men β=−0.201, R^2^= 0.051; women β=−0.113, R^2^ = 0.014; both p < 0.001), persisting after FMI adjustment (men β=−0.131, R^2^ = 0.159, p < 0.001; women β=−0.065, R^2^ = 0.046, p = 0.007).

**Table 3:** Multiple linear regression: standardized β and R^2^for body composition indices (ASMI, ALM/TLM) predicting FMI and HOMA-IR.

	Men			Women		
**Model**	**β**	**R²**	**p**	**β**	**R²**	**p**
FMI ← ASMI	+0.304	0.112	<0.001	+0.348	0.141	<0.001
FMI ← ALM/TLM	−0.205	0.069	<0.001	−0.255	0.089	<0.001
HOMA-IR ← ASMI	+0.104	0.021	<0.001	+0.080	0.008	<0.001
HOMA-IR ← ALM/TLM	−0.201	0.051	<0.001	−0.113	0.014	<0.001
HOMA-IR ← ALM/TLM + FMI adj.	−0.131	0.159	<0.001	−0.065	0.046	0.007

*Unweighted multiple linear regression. Adjusted for age, current smoking, alcohol ≥2 times/week, and regular physical activity.*

*ASMI = appendicular skeletal muscle mass index (ALM/height*^2^*, kg/m*^2^*ALM/TLM = appendicular lean mass to trunk lean mass ratio; FMI = fat mass index (kg/m*^2^*HOMA-IR = homeostasis model assessment of insulin resistance; β = standardized regression coefficient; R*^2^ *= coefficient of determination.*

### Low muscle mass and metabolic syndrome

Logistic regression results are presented in Table 4. ASMI-defined low muscle mass was inversely associated with MetS in men (Class I OR=0.496, 95% CI 0.388–0.636; Class II OR=0.413, 95% CI 0.275–0.621; both p < 0.001) and women (Class I OR=0.475, 95% CI 0.328–0.686; p < 0.001). The Class II ASMI estimate in women had extremely sparse cell counts (n = 4 No MetS, n = 1 MetS) and is unreliable. ALM/TLM low muscle mass showed dose-dependent increases in MetS odds: men Class I OR=1.730 (1.342–2.231), Class II OR=2.779 (2.074–3.722); women Class I OR=2.004 (1.567–2.563), Class II OR=2.701 (1.680–4.340; all p < 0.001). When modeled continuously, each 1-SD increase in ALM/TLM was associated with reduced MetS odds (men OR=0.648, 95% CI 0.568–0.739; women OR=0.676, 95% CI 0.609–0.750; both p < 0.001), whereas each 1-SD increase in ASMI was associated with increased MetS odds (men OR=1.532, 95% CI 1.352–1.737; women OR=1.587, 95% CI 1.433–1.757; both p < 0.001; S5 Table in S1 File). After additional FMI adjustment, the inverse ALM/TLM–MetS association persisted (men OR=0.761, 95% CI 0.661–0.876; women OR=0.800, 95% CI 0.714–0.898; both p < 0.001).

**Table 4:** Odds ratios from multivariable logistic regression for metabolic syndrome by low muscle mass category (n = 3,176).

Low muscle mass classification	No MetS	MetS	OR	95% CI
**Men – ASMI**				
No low muscle mass	398	372	1	—
Class I (≥6.155 to <7.031 kg/m²)	316	147	0.496	0.388–0.636
Class II (<6.155 kg/m²)	101	39	0.413	0.275–0.621
**Men – ALM/TLM**				
No low muscle mass	526	271	1	—
Class I (≥0.812 to <0.877)	220	193	1.730	1.342–2.231
Class II (<0.812)	69	94	2.779	2.074–3.722
**Women – ASMI**				
No low muscle mass	751	914	1	—
Class I (≥4.266 to <5.006 kg/m²)	83	49	0.475	0.328–0.686
Class II (<4.266 kg/m²) †	4	1	0.194	0.022–1.740
**Women – ALM/TLM**				
No low muscle mass	694	668	1	—
Class I (≥0.693 to <0.756)	119	231	2.004	1.567–2.563
Class II (<0.693)	25	65	2.701	1.680–4.340

*Reference: no low muscle mass within each sex. Adjusted for age, current smoking, alcohol ≥2 times/week, and regular physical activity.*

*† Women ASMI Class II: sparse cells (No MetS n = 4, MetS n = 1); estimate non-significant, interpret with caution.*

*ASMI = appendicular skeletal muscle mass index (ALM/height², kg/m²); ALM/TLM = appendicular lean mass to trunk lean mass ratio; OR = odds ratio; CI = confidence interval; MetS = metabolic syndrome.*

**Fig 2:**
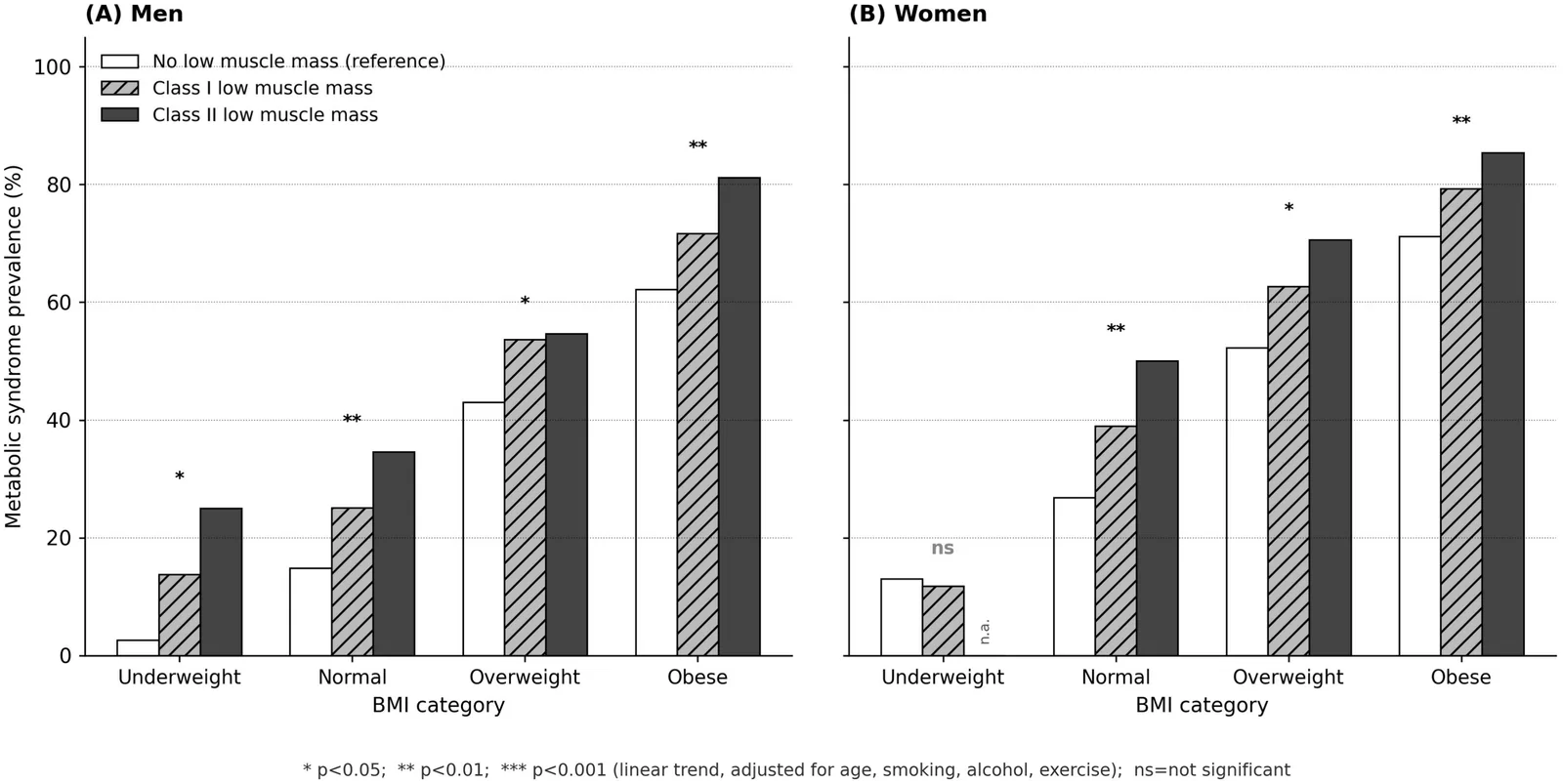
Metabolic syndrome prevalence (%) stratified by ALM/TLM low muscle mass category within BMI categories in adults aged ≥65 years. **(a)** Men. **(b)** Women. p-values are from survey-weighted linear trend tests adjusted for age, current smoking, alcohol consumption (≥2 times/week), and regular physical activity. BMI categories follow World Health Organization Asia–Pacific cut-offs. ALM/TLM = appendicular lean mass to trunk lean mass ratio; BMI = body mass index; MetS = metabolic syndrome.

Supplementary analyses using ALM/BMI and ALM/weight derived from the same reference group demonstrated positive MetS associations in both sexes (ALM/BMI Class II men OR=3.008, women OR=2.719; ALM/weight Class II men OR=6.841, women OR=3.613; S4 Table in S1 File). Stratification by BMI (Fig 2) showed significant linear trends in all four BMI strata in men and in normal, overweight, and obese strata in women. The underweight stratum in women showed a non-significant trend (p = 0.670), with limited statistical power given small cell counts in that subgroup. No consistent BMI-stratified linear trend was observed for ASMI.

### Simultaneous fat- and muscle-mass modeling

**Fig 3:**
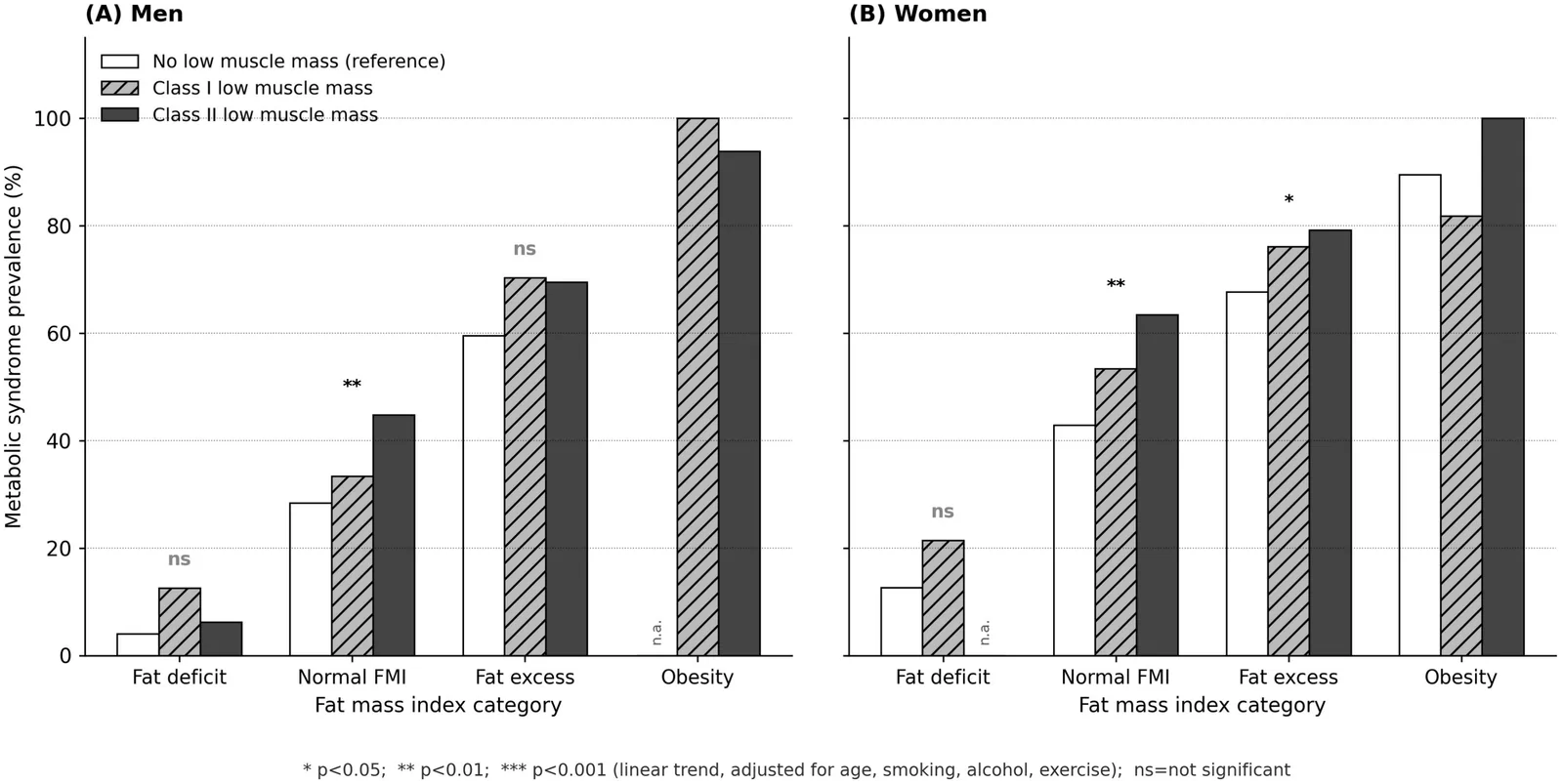
Metabolic syndrome prevalence (%) stratified by ALM/TLM low muscle mass category within fat mass index (FMI) categories in adults aged ≥65 years. **(a)** Men. **(b)** Women. p-values are from survey-weighted linear trend tests adjusted for age, current smoking, alcohol consumption (≥2 times/week), and regular physical activity. FMI categories: fat deficit (<3 kg/m^2^men, < 5 kg/m^2^women); normal (3–6/5–9 kg/m^2^fat excess (6–9/9–13 kg/m^2^); obesity (>9/ > 13 kg/m^2^). ALM/TLM = appendicular lean mass to trunk lean mass ratio; FMI = fat mass index; MetS = metabolic syndrome.

In the simultaneous model (Table 5), both FMI and ALM/TLM low muscle mass categories were independent MetS predictors. After FMI adjustment, ALM/TLM Class I (men OR=1.464, p = 0.007; women OR=1.518, p < 0.001) and Class II (men OR=2.028; women OR=2.085; both p < 0.001) remained significant. FMI stratification (Fig 3) showed significant linear trends in the normal fat mass stratum in both sexes (men: 28.4% → 33.4% → 44.8%, p < 0.001; women: 42.9% → 53.4% → 63.4%, p < 0.001).

**Table 5:** Simultaneous logistic regression: metabolic syndrome ~ FMI category + ALM/TLM low muscle mass (n = 3,176).

	Men		Women	
Variable	OR	95% CI	OR	95% CI
**FMI category (reference: normal FMI)**				
Fat deficit (Men < 3 / Women <5 kg/m²)	0.160	0.085–0.302	0.191	0.119–0.306
Fat excess (Men 6–9 / Women 9–13 kg/m²)	3.773	2.896–4.915	2.741	2.198–3.419
Obesity (Men > 9 / Women >13 kg/m²)	21.316	4.989–91.077	9.104	3.200–25.903
**ALM/TLM low muscle mass (reference: no low muscle mass)**				
Class I (≥0.812/0.693 to <0.877/0.756)	1.464	1.113–1.927	1.518	1.207–1.909
Class II (<0.812/0.693)	2.028	1.478–2.783	2.085	1.440–3.019

*Reference categories: normal FMI (Men 3–6, Women 5–9 kg/m* ^2^ *); no low muscle mass. Adjusted for age, current smoking, alcohol ≥2 times/week, and regular physical activity.*

*ALM/TLM = appendicular lean mass to trunk lean mass ratio; FMI = fat mass index (kg/m* ^2^ *OR = odds ratio; CI = confidence interval; MetS = metabolic syndrome.*

### Physical activity-stratified analysis

Physical activity (PA) was classified into two strata: sedentary (no regular physical activity; n = 1,403) and any regular physical activity (walking, moderate, or vigorous; n = 1,773). The positive direction of the ALM/TLM–MetS association was maintained in both strata and both sexes, with statistically significant associations across all Class II stratum–sex combinations and most Class I combinations (S6 Table in S1 File). Formal interaction testing showed no significant effect modification in either sex (men p for interaction = 0.231; women p = 0.945). ALM/TLM showed inverse associations with log-HOMA-IR across both PA strata in both sexes after FMI adjustment (S6 Table in S1 File). The mutually exclusive smoking–alcohol distribution is presented by sex in S9 Table in S1 File. Adjustment using this joint lifestyle variable did not change the direction or statistical significance of the association between ALM/TLM-defined low muscle mass and MetS in either sex; the interaction was not statistically significant in men and could not be reliably estimated in women. Additional adjustment for the percentage of energy derived from fat did not materially alter the ALM/TLM–MetS association in either sex (S10 Table in S1 File).

## Discussion

In this nationally representative cross-sectional study of older adults, the apparent association between low muscle mass and metabolic risk differed substantially according to the normalization strategy used. ASMI-defined low muscle mass was inversely associated with MetS in both sexes, whereas ALM/TLM-defined low muscle mass showed dose-dependent positive associations in both sexes. ALM/TLM was also inversely associated with HOMA-IR, and this association persisted after adjustment for measured fat mass index. Across adiposity strata, ALM/TLM showed a more directionally consistent association pattern than ASMI. These findings suggest that the normalization framework itself substantially influences the observed relationship between muscle mass and metabolic risk.

The inverse ASMI–MetS association is most plausibly explained by the structural properties of height-based normalization rather than by a true protective effect of low muscle mass. Height does not reflect body composition, and individuals with greater adiposity often have greater absolute lean mass as well. Consequently, individuals with obesity who have relatively low appendicular muscle mass for their body size may remain above the ASMI threshold, while individuals with lower adiposity and lower absolute lean mass may fall below it despite lower metabolic risk [5,6]. This interpretation is supported by the positive correlation between ASMI and FMI in both sexes (men r=+0.288; women r=+0.358), and by prior evidence that fat-adjusted lean mass indices consistently identify more individuals with low muscle mass than height-based ASMI, predominantly by reclassifying older adults with greater adiposity [17,18]. The within-sex prevalence pattern in this cohort provides additional supporting evidence. Even within women, where cut-off values were derived from the female reference population, ASMI-defined low muscle mass prevalence was paradoxically lower in the highest FMI quartile (2.7%) than in the lowest (14.0%). In contrast, ALM/TLM showed the opposite and more directionally consistent pattern (Q1: 28.8%; Q4: 48.1%; S7 Table in S1 File). This within-sex inverse gradient between adiposity and ASMI-defined low muscle mass is more consistent with adiposity-driven misclassification than with a genuine sex-specific biological difference. It also explains why the overall sex disparity in ASMI prevalence (43.9% in men vs. 8.0% in women) was substantially attenuated with ALM/TLM (57.7% vs. 40.2%).

By contrast, ALM/TLM showed consistently adverse associations with MetS and insulin resistance, including after adjustment for FMI (men β=−0.131, p < 0.001; women β=−0.065, p = 0.007). This does not establish that ALM/TLM is mechanistically superior but suggests that it may provide complementary information regarding the metabolic associations of relative muscle depletion. A possible explanation lies in the compositional properties of DXA-measured trunk lean mass. McCarthy et al. demonstrated, in a direct MRI–DXA cross-validation study (n = 475), that trunk lean mass comprised only approximately one-third skeletal muscle, with the remainder attributable to visceral lean tissues including the liver, kidneys, and heart [9]. Organs account for approximately 60–70% of resting metabolic rate despite representing less than 6% of body weight [10]. Given that the visceral organ fraction of TLM is not proportional to fat mass accumulation, TLM may behave differently from height as a denominator. The modest inverse correlations between ALM/TLM and FMI (men, r=−0.199; women, r=−0.243) indicate that ALM/TLM does not track adiposity in the same direction as ASMI; however, they do not establish independence from adiposity. Importantly, TLM should not be regarded as a fixed or biologically inert denominator. Central lean-mass distribution has itself been associated with adverse outcomes [19], indicating that the denominator may carry clinically relevant information; moreover, lean-mass measures vary with age and body size [20], and DXA-derived trunk lean mass may be influenced by hydration [21]. Consequently, a low ALM/TLM value may reflect lower ALM, higher TLM, or both. Component-level imaging and longitudinal validation are therefore required before ALM/TLM can be regarded as a stable or muscle-specific normalization index.

Several supplementary findings support the robustness of the primary result. First, all three non-height-based indices (ALM/TLM, ALM/BMI, and ALM/weight) showed positive associations with MetS, whereas ASMI did not, consistent with the interpretation that the paradox is specific to height-based normalization rather than to relative muscle mass assessment itself. Second, a trunk-to-leg lean mass ratio showed no independent metabolic contribution beyond shared variance with ALM/TLM (S8 Table in S1 File), suggesting both indices capture the same underlying body composition redistribution [19,22]. Third, physical activity-stratified analyses showed significant positive ALM/TLM–MetS associations in both sedentary and active strata in both sexes, with no significant interaction (men p = 0.231; women p = 0.945; S6 Table in S1 File), indicating that the association is not confined to physically inactive individuals.

These findings have direct relevance to the conceptual framework underlying current sarcopenic-obesity definitions. The ESPEN-EASO consensus statement on sarcopenic obesity recommends body composition assessment that can identify individuals with both excess adiposity and low lean mass [23]. Our results illustrate how height-based normalization may obscure relative muscle depletion in adults with high adiposity. ALM/TLM behaves more consistently across adiposity strata and warrants further evaluation in research settings, while its clinical utility remains to be established.

Our findings concern the metabolic associations of these indices and should be distinguished from their functional and clinical roles. ASMI remains the muscle-mass criterion in major sarcopenia frameworks [24,25], in which muscle strength and physical performance are assessed separately. An index showing limited or counterintuitive associations with metabolic risk in a given setting may still retain value as a measure of muscle quantity within a multidimensional sarcopenia assessment. Our findings therefore do not diminish the established role of ASMI within current frameworks. Rather, they suggest that ALM/TLM may provide complementary information regarding metabolic-risk associations and should be evaluated alongside, rather than as a replacement for, height-based indices.

The present study has several limitations. First, the study relied on muscle mass alone without concurrent assessment of muscle strength or physical performance as required by current consensus definitions of sarcopenia [24,25]. This study is therefore more accurately characterized as a metabolically informative body composition index study than a sarcopenia diagnostic validation study, and the ALM/TLM categories should not be interpreted as clinically validated sarcopenia thresholds. Whether ALM/TLM offers any advantage over established indices for predicting functional outcomes such as weakness, mobility limitation, falls, or mortality remains unknown and requires prospective investigation. Second, the cross-sectional study design precludes causal inference; the observed associations are consistent with the proposed interpretation but do not establish mechanistic proof. Third, the study cohort was restricted to participants who completed a DXA examination, and the survey-weighted MetS prevalence in this DXA subsample (men 42.5%; women 57.1%) was somewhat higher than published estimates for the general population in this age range from comparable survey periods. This may reflect selection factors related to DXA participation. Fourth, medication use affecting muscle metabolism (e.g., corticosteroids, hormonal agents) was not available in this dataset and could not be controlled for. Fifth, although supplementary analyses of ALM/BMI and ALM/weight are presented, we did not conduct a comprehensive comparison of ALM/TLM against all currently proposed normalization strategies [23], and the relative performance of each index for functional endpoints, including handgrip strength and gait speed, remains untested. Sixth, DXA-measured TLM does not differentiate visceral organs from axial skeletal muscle mass; direct validation of TLM stability using MRI or CT-based organ volumetry across age groups, disease states, and adiposity strata is needed before its premise as a biologically stable normalizer can be confirmed. Seventh, physical activity was self-reported and categorized as a four-level ordinal variable without information on activity duration, intensity quantification, or type (resistance vs. aerobic). Therefore, misclassification of activity level and limited statistical power in the smaller moderate and vigorous strata may have precluded detection of genuine effect modification, and the non-significant interaction tests should be interpreted with caution. Single-measurement HOMA-IR may also introduce biological variation [26]. Finally, the study sample comprised exclusively older Korean adults, and the sex-specific ALM/TLM and ASMI cut-offs were derived from a Korean young-adult reference group. Because body composition, adiposity distribution, and sarcopenia prevalence vary across ethnic groups, the absolute cut-off values and magnitude of the observed associations may not generalize to other populations. External validation in independent populations with different ethnic backgrounds is required before these cut-offs or their clinical interpretation can be generalized.

## Conclusions

In a nationally representative sample of older adults, height-based normalization produced a paradoxical inverse association between ASMI-defined low muscle mass and MetS, whereas ALM/TLM showed directionally consistent positive associations across adiposity strata and after adjustment for measured fat mass index. These findings indicate that normalization strategies substantially affect the apparent metabolic associations of low muscle mass. ALM/TLM showed more directionally consistent associations with MetS across adiposity strata and warrants further evaluation as a research candidate. Whether it predicts clinically meaningful outcomes better than ASMI remains unknown, and it should not currently be used for clinical classification or decision-making. External validation in cohorts with functional measures and prospective clinical outcomes is required.

## Supporting information

S1 File Supplementary Tables S1–S10. Young reference group characteristics (S1 Table); sex-specific cut-off derivation (S2 Table); survey-weighted baseline characteristics (S3 Table); odds ratios across four normalization strategies (S4 Table); 1-SD continuous models (S5 Table); physical activity-stratified analyses (S6 Table); within-sex low muscle mass prevalence by FMI category (S7 Table); and ALM/TLM and trunk-to-leg lean mass ratio comparison (S8 Table). Joint distribution of current smoking and alcohol consumption ≥2 times/week and lifestyle-adjusted associations between ALM/TLM class and metabolic syndrome (S9 Table); and dietary-fat sensitivity analysis of the ALM/TLM–metabolic syndrome association (S10 Table).
(DOCX)

S1 Fig Overlap between current smoking and alcohol consumption ≥2 times/week, by sex. (a) Men. (b) Women. Numbers indicate participants in each mutually exclusive category. Participants with complete smoking and alcohol data: men, n = 1,372; women, n = 1,802.
(TIF)

S1 Checklist STROBE checklist for cross-sectional studies. (DOCX)
