# Supplementary material for: Appendicular-to-trunk lean mass ratio resolves the height-based normalization paradox in low muscle mass and metabolic syndrome: A population-based cross-sectional study
Source: PLoS One. 2026 Aug 25;21(8):e0357017. doi: 10.1371/journal.pone.0357017 (PMC13505910; doi:10.1371/journal.pone.0357017)
Supplement: Supplementary file 1 [file pone.0357017.s004.pdf]

Comments:

The author has presented thoughtful ideas, and the work is appreciated for its interpretation and results. However, there are still several areas that require improvement. The manuscript would benefit from further refinement in clarity, structure, and depth of analysis to strengthen its overall quality and impact.

Abstract: (line 29-33) **ASMI validity concern**: ASMI shows strong correlations with adiposity (BMI/FMI) and paradoxical protective associations with metabolic syndrome, suggesting it may not represent muscle mass but a body-size/adiposity proxy. Interpretation should be substantially revised.

Introduction: (line 48-50) Please Include recent high-quality journal references in the Introduction to strengthen background and support study rationale

Study Designed: The study population should be clearly divided into mutually exclusive categories (non-users, single exposure (like only alcohol or smokers), and dual exposure groups alcohol +smokers and heavy or small quantity) with sample sizes and percentages.

Results: Do you have data on participants' dietary habits? If yes, could dietary habits (fried/oil-based versus non-fried food consumption) be systematically classified and reported alongside smoking and alcohol status?

Figures: Please include a Venn diagram to clearly show overlap between exposure groups (smoking, alcohol, and dietary habits) with proper population stratification.
