# Supplementary material for: Appendicular-to-trunk lean mass ratio resolves the height-based normalization paradox in low muscle mass and metabolic syndrome: A population-based cross-sectional study
Source: PLoS One. 2026 Aug 25;21(8):e0357017. doi: 10.1371/journal.pone.0357017 (PMC13505910; doi:10.1371/journal.pone.0357017)
Supplement: Supplementary file 2 [file pone.0357017.s001.docx]

**Supplementary Table S1.** Young reference group characteristics (aged 20–39 years; KNHANES 2008–2010)

| **Variable** | **Men (n=1,768)** | **Women (n=1,959)** | **p value** |
| --- | --- | --- | --- |
| **Demographics** |  |  |  |
| Age (years) | 31.2 ± 5.4 | 30.9 ± 5.7 | 0.061 |
| Height (cm) | 173.7 ± 5.8 | 160.5 ± 5.5 | <0.001 |
| Weight (kg) | 72.5 ± 11.1 | 56.4 ± 9.1 | <0.001 |
| BMI (kg/m²) | 24.0 ± 3.3 | 21.9 ± 3.3 | <0.001 |
| **Body composition – DXA** |  |  |  |
| ALM (g) | 23,877 ± 3,189 | 14,718 ± 2,233 | <0.001 |
| TLM (g) | 25,407 ± 3,210 | 17,999 ± 2,346 | <0.001 |
| Limb BMC (g) | 1,367 ± 200 | 942 ± 132 | <0.001 |
| Trunk BMC (g) | 685 ± 118 | 543 ± 90 | <0.001 |
| ALM/height² – ASMI (kg/m²) | 7.906 ± 0.876 | 5.745 ± 0.740 | <0.001 |
| ALM/TLM | 0.942± 0.065 | 0.818 ± 0.062 | <0.001 |
| FMI (kg/m²) | 5.2 ± 2.0 | 6.8 ± 2.1 | <0.001 |
| Body fat (%) | 21.6 ± 6.0 | 31.5 ± 5.4 | <0.001 |

*Data are mean ± SD; p-values by independent t-test.*

*Bone mineral content (BMC) is reported separately by DXA and is not included in lean soft tissue compartments.*

*ALM, appendicular lean mass, defined as the sum of arm and leg lean soft tissue mass on DXA; TLM, trunk lean mass, defined as trunk lean soft tissue mass on DXA; ASMI, appendicular skeletal muscle index; FMI, fat mass index.*

**Supplementary Table S2.** Sex-specific cut-off values for low muscle mass indices derived from the young reference group

| **Index** | **Men mean ± SD** | **Class I** | **Class II** | **Women mean ± SD** | **Class I** | **Class II** |
| --- | --- | --- | --- | --- | --- | --- |
| ASMI (kg/m²) | 7.906 ± 0.876 | < 7.031 | < 6.155 | 5.745 ± 0.740 | < 5.006 | < 4.266 |
| ALM/TLM | 0.942± 0.065 | < 0.877 | < 0.812 | 0.818 ± 0.062 | < 0.756 | < 0.693 |

*Class I (low muscle mass): 1 SD below the sex-specific young-adult mean; Class II (very low muscle mass): 2 SD below the mean.*

*ASMI = appendicular lean mass/height² (kg/m²); ALM/TLM = appendicular lean mass/trunk lean mass (both BMC-excluded).*

**Supplementary Table S3.** Survey-weighted baseline characteristics of study participants aged ≥65 years by sex (KNHANES 2008–2010; n=3,176)

| **Variable** | **Men (n=1,373)** | **Women (n=1,803)** |
| --- | --- | --- |
| **Demographics** |  |  |
| Age (years) | 71.6 ± 5.1 | 72.4 ± 5.3 |
| Height (cm) | 164.9 ± 5.8 | 150.9 ± 5.8 |
| Weight (kg) | 63.0 ± 9.3 | 55.3 ± 8.9 |
| BMI (kg/m²) | 23.1 ± 2.9 | 24.2 ± 3.3 |
| Waist circumference (cm) | 84.6 ± 8.9 | 83.6 ± 9.5 |
| **Body composition – DXA** |  |  |
| ALM (kg) | 19.4 ± 2.7 | 13.5 ± 1.9 |
| TLM (kg) | 22.6 ± 3.0 | 17.4 ± 2.3 |
| ALM/height² – ASMI (kg/m²) | 7.16 ± 0.78 | 5.89 ± 0.65 |
| ALM/TLM | 0.86 ± 0.07 | 0.78 ± 0.07 |
| FMI (kg/m²) | 5.2 ± 1.7 | 8.3 ± 2.3 |
| Body fat (%) | 22.7 ± 5.4 | 34.4 ± 5.7 |
| **Metabolic parameters** |  |  |
| Systolic BP (mmHg) | 130.9 ± 17.7 | 133.3 ± 17.9 |
| Diastolic BP (mmHg) | 77.6 ± 9.9 | 77.3 ± 9.8 |
| Fasting plasma glucose (mg/dL) | 105.1 ± 26.8 | 104.8 ± 27.1 |
| Total cholesterol (mg/dL) | 180.7 ± 34.5 | 199.9 ± 36.6 |
| HDL cholesterol (mg/dL) | 48.1 ± 12.3 | 49.7 ± 11.7 |
| Triglycerides (mg/dL) | 145.8 ± 100.0 | 149.1 ± 86.0 |
| HOMA-IR | 2.5 ± 1.7 | 3.0 ± 2.9 |
| Metabolic syndrome (%) | 42.5 | 57.1 |

*Survey-weighted mean ± SD or weighted prevalence (%). Sampling weights (wt_tot), stratification variables (kstrata), and primary sampling units (psu) are applied.*

*Weighted MetS prevalence: 42.5% (men) and 57.1% (women); cf. unweighted 40.6% and 53.5%, reflecting the complex survey design.*

*ASMI = appendicular skeletal muscle mass index (ALM/height², kg/m²); ALM/TLM = appendicular lean mass to trunk lean mass ratio; FMI = fat mass index (kg/m²); HOMA-IR = homeostasis model assessment of insulin resistance; HDL = high-density lipoprotein; MetS = metabolic syndrome; BMI = body mass index; DXA = dual-energy X-ray absorptiometry; SD = standard deviation.*

**Supplementary Table S4.** Odds ratios for metabolic syndrome across four ALM normalization strategies: ASMI, ALM/TLM, ALM/BMI, and ALM/weight (adjusted for age, current smoking, alcohol ≥2 times/week, and regular physical activity; n=3,176)

| **Index / Sarcopenia class** | **No MetS** | **MetS** | **OR** | **95% CI** |
| --- | --- | --- | --- | --- |
| **Men – ASMI** | | | | |
| No low muscle mass (reference) | 398 | 372 | 1 | — |
| Class I (≥6.155 to < 7.031 kg/m²) | 316 | 147 | 0.496 | 0.388–0.636 |
| Class II (< 6.155 kg/m²) | 101 | 39 | 0.413 | 0.275–0.621 |
| **Men – ALM/TLM** | | | | |
| No low muscle mass | 526 | 271 | 1 | — |
| Class I (≥0.812 to < 0.877) | 220 | 193 | 1.730 | 1.342–2.231 |
| Class II (< 0.812) | 69 | 94 | 2.779 | 2.074–3.722 |
| **Men – ALM/BMI** | | | | |
| No low muscle mass | 327 | 136 | 1 | — |
| Class I (≥0.767 to < 0.884) | 356 | 267 | 1.870 | 1.442–2.425 |
| Class II (< 0.767) | 132 | 155 | 3.008 | 2.190–4.131 |
| **Men – ALM/weight** | | | | |
| No low muscle mass | 604 | 250 | 1 | — |
| Class I (≥0.272 to < 0.302) | 179 | 220 | 2.929 | 2.280–3.763 |
| Class II (< 0.272) | 32 | 88 | 6.841 | 4.414–10.602 |
| **Women – ASMI** | | | | |
| No low muscle mass | 751 | 914 | 1 | — |
| Class I (≥4.266 to < 5.006 kg/m²) | 83 | 49 | 0.475 | 0.328–0.686 |
| Class II (< 4.266 kg/m²) † | 4 | 1 | 0.194 | 0.022–1.740 |
| **Women – ALM/TLM** | | | | |
| No low muscle mass | 694 | 668 | 1 | — |
| Class I (≥0.693 to < 0.756) | 119 | 231 | 2.004 | 1.567–2.563 |
| Class II (< 0.693) | 25 | 65 | 2.701 | 1.680–4.340 |
| **Women – ALM/BMI** | | | | |
| No low muscle mass | 363 | 255 | 1 | — |
| Class I (≥0.508 to < 0.592) | 333 | 439 | 1.878 | 1.515–2.328 |
| Class II (< 0.508) | 142 | 270 | 2.719 | 2.092–3.534 |
| **Women – ALM/weight** | | | | |
| No low muscle mass | 634 | 498 | 1 | — |
| Class I (≥0.213 to < 0.238) | 163 | 348 | 2.704 | 2.167–3.373 |
| Class II (< 0.213) | 41 | 118 | 3.613 | 2.482–5.259 |

*Survey-weighted logistic regression. All cut-offs derived by the same method (1 SD = Class I; 2 SD = Class II below the sex-specific mean of the young reference group, aged 20–39 years; n=3,727).*

*ALM/BMI cut-offs: Class I <0.884 (men) / <0.592 (women); Class II <0.767 / <0.508. ALM/weight cut-offs: Class I <0.302 / <0.238; Class II <0.272 / <0.213.*

*† Women ASMI Class II: sparse cells; estimate unreliable.*

**Supplementary Table S5.** Odds ratios for metabolic syndrome per 1-SD change in ASMI, ALM/TLM, and FMI; older adults aged ≥65 years (KNHANES 2008–2010; n=3,176).

| **Index** | **OR (Men)** | **95% CI (Men)** | **OR (Women)** | **95% CI (Women)** |
| --- | --- | --- | --- | --- |
| **A. Unadjusted for FMI** | | | | |
| ASMI (per 1-SD↑) | 1.532 | 1.352–1.737 | 1.587 | 1.433–1.757 |
| ALM/TLM (per 1-SD↑) | 0.648 | 0.568–0.739 | 0.676 | 0.609–0.750 |
| FMI (per 1-SD↑) | 2.913 | 2.510–3.382 | 2.750 | 2.405–3.145 |
| **B. ALM/TLM additionally adjusted for FMI** | | | | |
| ALM/TLM (per 1-SD↑, FMI adj) | 0.761 | 0.661–0.876 | 0.800 | 0.714–0.898 |

*OR = odds ratio; CI = confidence interval. ASMI = appendicular skeletal muscle mass index (ALM/height²); ALM/TLM = appendicular lean mass to trunk lean mass ratio; FMI = fat mass index. Each index is standardized to 1 SD within sex. All models adjusted for age, current smoking, alcohol consumption (≥2 times/week), and regular physical activity. The FMI-adjusted model includes FMI as an additional covariate. All p<0.001.*

**Supplementary Table S6.** Physical activity-stratified associations of ALM/TLM Class I low muscle mass with metabolic syndrome (Panel A) and insulin resistance (Panel B) in older adults aged ≥65 years (KNHANES 2008–2010; n=3,176).

**A. Metabolic syndrome: OR by PA stratum (survey-weighted)**

|  | **Sedentary**  **(no regular physical activity)** | | **Any regular physical activity** | |
| --- | --- | --- | --- | --- |
|  | **Men** | **Women** | **Men** | **Women** |
| **Class I low muscle mass** |  |  |  |  |
| *OR (95% CI)* | 2.084  (1.357–3.200) | 1.778  (1.316–2.402) | 1.480  (1.077–2.034) | 1.673  (1.225–2.284) |
| *p value* | <0.001 | <0.001 | 0.016 | 0.001 |
| **Class II low muscle mass** |  |  |  |  |
| *OR (95% CI)* | 2.651  (1.644–4.273) | 3.373  (2.006–5.671) | 3.013  (2.079–4.366) | 3.164  (1.901–5.265) |
| *p value* | <0.001 | <0.001 | <0.001 | <0.001 |
| *p for interaction (LRT, 2 df)* | 0.231 | | 0.945 | |

**B. Insulin resistance: ALM/TLM β for log-HOMA-IR by PA stratum (survey-weighted, FMI-adjusted)**

|  | **Sedentary (no regular physical activity)** | | **Any regular physical activity** | |
| --- | --- | --- | --- | --- |
|  | **Men** | **Women** | **Men** | **Women** |
| Standardized β | −0.067 | −0.042 | −0.081 | −0.037 |
| R² | 0.231 | 0.186 | 0.252 | 0.178 |
| p value | 0.001 | 0.007 | <0.001 | 0.023 |

*PA classification: Sedentary = no regular PA of any kind; Any regular PA= walking, moderate-intensity, or vigorous-intense activity.*

*Panel A: Survey-weighted logistic regression with PSU cluster-robust sandwich standard errors. Reference = no low muscle mass. Adjusted for age, current smoking, and alcohol ≥2 times/week. Single-PSU strata: 4–10% (vs. 31–40% for 4-level classification); small-sample HC correction applied.*

*p for interaction: likelihood-ratio test comparing a main-effects model (ALM/TLM class + physical activity stratum + covariates) against a model additionally including ALM/TLM class × physical activity interaction terms (2 df, corresponding to Class I × PA and Class II × PA).*

*Panel B: Survey-weighted linear regression with PSU cluster-robust standard errors. Adjusted for FMI, age, current smoking, and alcohol ≥2 times/week. β standardized per 1 SD of ALM/TLM within each stratum.*

*ALM/TLM = appendicular lean mass to trunk lean mass ratio; OR = odds ratio; CI = confidence interval; MetS = metabolic syndrome; HOMA-IR = homeostasis model assessment of insulin resistance; β = standardized regression coefficient; PA = physical activity.*

**Supplementary Table S7.** Low muscle mass prevalence (%) by FMI category: comparison of ASMI and ALM/TLM in older adults aged ≥65 years (KNHANES 2008–2010; n=3,176).

| **FMI category** | | **ASMI** | | **ALM/TLM** | |
| --- | --- | --- | --- | --- | --- |
|  | **n** | **Class I+II (%)** | **Class II (%)** | **Class I+II (%)** | **Class II (%)** |
| **Men** | | | | | |
| Fat deficit ( <3 kg/m²) | 204 | 60.8 | 21.6 | 39.2 | 11.8 |
| Normal (3–6 kg/m²) | 830 | 44.8 | 8.7 | 58.4 | 21.1 |
| Fat excess (6–9 kg/m²) | 316 | 32.9 | 7.6 | 69.3 | 31.3 |
| Obesity ( >9 kg/m²) | 23 | 13.0 | 0.0 | 82.6 | 47.8 |
| **Women** | | | | | |
| Fat deficit (<5 kg/m²) | 191 | 15.2 | 0.5 | 23.6 | 2.6 |
| Normal (5–9 kg/m²) | 1063 | 8.7 | 0.4 | 33.4 | 8.7 |
| Fat excess (9–13 kg/m²) | 517 | 3.3 | 0.0 | 48.2 | 12.6 |
| Obesity (>13 kg/m²) | 32 | 0.0 | 0.0 | 46.9 | 25.0 |

*ASMI = appendicular skeletal muscle mass index (ALM/height², kg/m²); ALM/TLM = appendicular lean mass to trunk lean mass ratio; FMI = fat mass index (kg/m²).*

**Supplementary Table S8.** Relationship between ALM/TLM and trunk-to-leg lean mass ratio (TLR; Ding et al., 2024): correlation and metabolic association comparison in older adults aged ≥65 years.

|  | **ALM/TLM (per 1-SD decrease)** | | **TLR (per 1-SD increase)** | | **p value** |
| --- | --- | --- | --- | --- | --- |
|  | **Men** | **Women** | **Men** | **Women** |  |
| **A. Pearson correlation: ALM/TLM vs TLR** | | | | | |
| r | −0.945 | −0.955 | — | — | <0.001 (both) |
| **B. MetS OR (IDF definition), per 1-SD change** | | | | | |
| OR (95% CI) | 1.490  [1.291–1.719] | 1.449  [1.301–1.614] | 1.279  [1.121–1.460] | 1.306  [1.178–1.448] | <0.001 (all) |
| **C. MetS OR (NCEP definition), per 1-SD change** | | | | | |
| OR (95% CI) | 1.550  [1.370–1.753] | 1.478  [1.334–1.637] | 1.403  [1.245–1.580] | 1.385  [1.252–1.533] | <0.001 (all) |
| **D. log-HOMA-IR β after FMI adjustment, per 1-SD change** | | | | | |
| β (95% CI) | +0.074  [+0.050–+0.098] | +0.040  [+0.018–+0.062] | +0.065  [+0.042–+0.089] | +0.043  [+0.021–+0.065] | <0.001 (all) |
| **E. Simultaneous model: both indices entered as predictors of log-HOMA-IR** | | | | | |
| ALM/TLM β | +0.294*** | +0.205*** | — | — | <0.001 (both) |
| TLR β | −0.181*** | −0.122** | — | — | <0.001 / 0.002 |

*MetS models adjusted for age, current smoking, alcohol ≥2 times/week, and regular physical activity. Reported per 1 SD decrease in ALM/TLM or 1 SD increase in TLR, so that both indicate worse body composition.*

*Section E (simultaneous model): both indices entered together as predictors of log-transformed HOMA-IR with the same covariates.*

*** p<0.01; *** p<0.001.*

*OR = odds ratio; CI = confidence interval; ASMI = appendicular skeletal muscle mass index (ALM/height², kg/m²); FMI = fat mass index (kg/m²); HOMA-IR = homeostasis model assessment of insulin resistance; β = standardized regression coefficient; DXA = dual-energy X-ray absorptiometry; SD = standard deviation.*

**S9 Table. Smoking × alcohol × ALM/TLM class interaction analysis (sex-stratified complex-samples logistic regression).**

**(a) Four-category lifestyle exposure — sex-stratified cell counts (n); % of total**

| **Lifestyle category** | **Men (n=1,372)** | **Women (n=1,802)** | **Total (n=3,174)** | **% of total** |
| --- | --- | --- | --- | --- |
| Neither exposure | 670 | 1,621 | 2,291 | 72.2 |
| Alcohol ≥2 times/week only | 350 | 90 | 440 | 13.9 |
| Current smoking only | 191 | 83 | 274 | 8.6 |
| Both exposures | 161 | 8 | 169 | 5.3 |
| Total | 1,372 | 1,802 | 3,174 | 100.0 |

Unweighted cell counts. Total N = 3,174 reflects 2 participants with missing smoking or alcohol data. The women × “both” cell is small (n = 8).

**(b) Sex-stratified interaction models — Type III Wald F test (men)**

| **Source** | **F** | **num df** | **denom df** | **P value** |
| --- | --- | --- | --- | --- |
| ALM/TLM class | 15.86 | 2 | 414 | <0.001 |
| Smoking–alcohol category | 2.40 | 3 | 413 | 0.068 |
| Age | 4.01 | 1 | 415 | 0.046 |
| Regular physical activity | 1.13 | 1 | 415 | 0.288 |
| ALM/TLM class × smoking–alcohol | 2.08 | 6 | 410 | 0.054 |

**Women:** the interaction model did not converge (quasi-complete separation arising from the small women × “both” cell, n = 8); the main-effects model is reported in panel (c).

**(c) Main-effects models — adjusted odds ratios (Normal / Neither as reference)**

| **Contrast** | **Men, OR (95% CI)** | **Women, OR (95% CI)** |
| --- | --- | --- |
| ALM/TLM Class I vs Normal | 1.95 (1.44–2.65) | 1.51 (1.16–1.98) |
| ALM/TLM Class II vs Normal | 2.43 (1.76–3.36) | 2.80 (1.79–4.40) |
| Alcohol ≥2/week only vs Neither | 0.90 (0.65–1.25) | 0.80 (0.45–1.42) |
| Smoking only vs Neither | 0.86 (0.56–1.33) | 0.81 (0.46–1.41) |
| Both vs Neither | 0.48 (0.31–0.75) | 0.64 (0.12–3.38) |
| Wald F for ALM/TLM class | F=17.01, P<0.001 | F=12.29, P<0.001 |

**Legend.** Complex-samples logistic regression with strata (kstrata), primary sampling units (psu), and analytic weight (wt_tot); sex-stratified via /DOMAIN VARIABLE=sex. Outcome: NCEP metabolic syndrome (0 = No / 1 = Yes). Lifestyle categories: neither exposure (not a current smoker and alcohol <2 times/week); alcohol ≥2 times/week only (not a current smoker and alcohol ≥2 times/week); current smoking only (current smoker and alcohol <2 times/week); both exposures (current smoker and alcohol ≥2 times/week). ALM/TLM class based on the bone-mineral-content–excluded definition, with sex-specific cut-offs from the young-adult reference group (Class I <0.877 in men and <0.756 in women; Class II <0.812 and <0.693). Models adjusted for age and regular physical activity. Reference categories set via /ODDSRATIOS FACTOR=[factor(LOW)]. The main-effects estimates were obtained from models containing the four-category joint smoking–alcohol variable in place of the separate smoking and alcohol covariates used in Table 4 and should therefore be interpreted as results from a distinct lifestyle-adjusted model. OR = odds ratio; CI = confidence interval.

**S10 Table. Dietary-fat sensitivity analysis: ALM/TLM association with metabolic syndrome, with and without adjustment for percentage of energy from fat.**

**(a) ALM/TLM odds ratios**

| **Contrast** | **Model A (no dietary fat) OR (95% CI)** | **Model B (+ fat % of energy) OR (95% CI)** |
| --- | --- | --- |
| Men, Class I vs Normal | 1.907 (1.403–2.593) | 1.911 (1.406–2.597) |
| Men, Class II vs Normal | 2.398 (1.745–3.296) | 2.418 (1.761–3.322) |
| Women, Class I vs Normal | 1.517 (1.160–1.984) | 1.516 (1.159–1.984) |
| Women, Class II vs Normal | 2.804 (1.789–4.393) | 2.799 (1.789–4.381) |

**(b) Wald F test for percentage of energy from fat (Model B)**

| **Predictor** | **Men** | **Women** |
| --- | --- | --- |
| Fat % of energy: F (1, 415) | 0.58 | 0.24 |
| Fat % of energy: P value | 0.445 | 0.627 |

**Legend.** Sensitivity analysis restricted to participants with complete 24-hour dietary recall and covariate data. Percentage of energy from fat = dietary fat (g) × 9 kcal/g ÷ total energy intake (kcal) × 100, from the 24-hour recall. Both models used the same analytic sample, the primary-analysis sampling weight (wt_tot), strata, and primary sampling units; the bone-mineral-content–excluded ALM/TLM definition and cut-offs; and covariates (age, current smoking, alcohol ≥2 times/week, regular physical activity). Model B additionally included percentage of energy from fat. Sex-stratified complex-samples logistic regression. OR = odds ratio; CI = confidence interval.
