# Supplementary material for: Appendicular-to-trunk lean mass ratio resolves the height-based normalization paradox in low muscle mass and metabolic syndrome: A population-based cross-sectional study
Source: PLoS One. 2026 Aug 25;21(8):e0357017. doi: 10.1371/journal.pone.0357017 (PMC13505910; doi:10.1371/journal.pone.0357017)
Supplement: Supplementary file 3 [file pone.0357017.s003.docx]

STROBE Statement—Checklist of items that should be included in reports of ***cross-sectional studies***

|  | Item No | Recommendation | Page No |
| --- | --- | --- | --- |
| **Title and abstract** | 1 | (*a*) Indicate the study’s design with a commonly used term in the title or the abstract | Title (line 2): “… A population-based cross-sectional study”  Abstract (lines 18–38): KNHANES 2008–2010 secondary analysis stated. |
|  |  | (*b*) Provide in the abstract an informative and balanced summary of what was done and what was found | Abstract (lines 18–38): unstructured prose abstract, ≤300 words covering background, methods, results, and conclusion. |
| Introduction | | | |
| Background/rationale | 2 | Explain the scientific background and rationale for the investigation being reported | Lines 44–66 (Introduction paragraphs 1–2): skeletal muscle as the principal site of insulin-stimulated glucose disposal; limitations of height-based and weight/BMI-based normalization indices. |
| Objectives | 3 | State specific objectives, including any prespecified hypotheses | Lines 67–79: “We therefore explored ALM normalized to trunk lean mass (TLM) as an alternative denominator strategy … We hypothesized that ALM/TLM would present a more metabolically coherent association pattern than ASMI in older adults.” |
| Methods | | | |
| Study design | 4 | Present key elements of study design early in the paper | Line 93: “This cross-sectional study used data from the Korea National Health and Nutrition Examination Survey (KNHANES) 2008–2010.” |
| Setting | 5 | Describe the setting, locations, and relevant dates, including periods of recruitment, exposure, follow-up, and data collection | Lines 93–97: KNHANES described as a nationally representative survey using clustered, multistage, stratified probability sampling, with annual rolling samples spanning 2008–2010. Ethics statement on lines 81–90. |
| Participants | 6 | (*a*) Give the eligibility criteria, and the sources and methods of selection of participants | Lines 93–106: Primary analysis cohort (n=3,176) and exclusion criteria; medication use disclosure; young reference group derivation (n=3,727; aged 20–39 years; metabolic disease excluded). |
| Variables | 7 | Clearly define all outcomes, exposures, predictors, potential confounders, and effect modifiers. Give diagnostic criteria, if applicable | Lines 108–134: “DXA measurements and body composition” (L108); “Low muscle mass definitions” (L116) including Class I/II thresholds; “Metabolic syndrome and insulin resistance” (L123) including NCEP-ATP III + population-specific waist cut-offs, HOMA-IR formula, and FMI categories. |
| Data sources/ measurement | 8* | For each variable of interest, give sources of data and details of methods of assessment (measurement). Describe comparability of assessment methods if there is more than one group | Lines 109–115 (DXA equipment: Hologic QDR 4500A; Discovery v13.1); lines 123–134 (MetS NCEP-ATP III definition; HOMA-IR formula; FMI category derivation referenced to DXA reference data). |
| Bias | 9 | Describe any efforts to address potential sources of bias | Lines 93–106 (cohort exclusions); lines 144–150 (survey-weighted analysis incorporating sampling weights, strata, and PSU); lines 346–371 (Discussion limitations: selection bias from DXA participation; residual confounding due to missing medication data; single HOMA-IR measurement). |
| Study size | 10 | Explain how the study size was arrived at | Lines 93–101: study size (n=3,176) determined by all eligible KNHANES 2008–2010 participants aged ≥65 years with complete DXA and laboratory data after the listed exclusions; no a priori power calculation. |
| Quantitative variables | 11 | Explain how quantitative variables were handled in the analyses. If applicable, describe which groupings were chosen and why | Lines 117–121 (low muscle mass cut-offs at 1 SD and 2 SD below young-adult mean for ASMI and ALM/TLM); lines 131–134 (FMI category thresholds and rationale); line 141 (HOMA-IR log transformation due to right skew). |
| Statistical methods | 12 | (*a*) Describe all statistical methods, including those used to control for confounding | Lines 137–155: SPSS Complex Samples (CSLOGISTIC) with cluster-robust SEs; covariates listed (age, smoking, alcohol, physical activity). |
|  |  | (*b*) Describe any methods used to examine subgroups and interactions | Lines 148–155: BMI- and FMI-stratified analyses with linear trend tests; physical activity-stratified analyses (sedentary vs. active) with formal interaction testing. |
|  |  | (*c*) Explain how missing data were addressed | Lines 93–101: complete-case analysis after exclusion of participants with missing DXA or laboratory data. Lines 100–101: medication use was unavailable in the dataset and could not be controlled for (also Discussion lines 346–371). |
|  |  | (*d*) If applicable, describe analytical methods taking account of sampling strategy | Lines 144–150: sampling weights (wt_tot), strata (kstrata), and primary sampling units (psu) incorporated using KNHANES analytic guidelines for inferential analyses. |
|  |  | (*e*) Describe any sensitivity analyses | Lines 148–155: 1-SD continuous models with and without FMI adjustment (S5 Table); ALM/BMI and ALM/weight comparisons (S4 Table); physical activity stratification (S6 Table); within-sex prevalence by FMI category (S7 Table); trunk-to-leg lean mass ratio comparison (S8 Table); all in S1 File. |
| Results | | | |
| Participants | 13* | (a) Report numbers of individuals at each stage of study—eg numbers potentially eligible, examined for eligibility, confirmed eligible, included in the study, completing follow-up, and analysed | Lines 93–106 and 157–165: cohort numbers (n=3,176 analytic; n=3,727 reference) and exclusion criteria stated. |
|  |  | (b) Give reasons for non-participation at each stage | n/a – individual-level reasons for non-participation are not separately documented for KNHANES rolling samples; cohort exclusion logic is reported (lines 93–106). |
|  |  | (c) Consider use of a flow diagram | n/a – given the simple inclusion logic, a flow diagram was not used; cohort numbers are stated narratively. |
| Descriptive data | 14* | (a) Give characteristics of study participants (eg demographic, clinical, social) and information on exposures and potential confounders | Lines 157–165 (“Baseline characteristics”); Table 1 embedded in the manuscript directly after this paragraph; S3 Table in S1 File (survey-weighted estimates). |
|  |  | (b) Indicate number of participants with missing data for each variable of interest | Lines 93–101: missing DXA or laboratory data was an exclusion criterion (complete-case analytic cohort). |
| Outcome data | 15* | Report numbers of outcome events or summary measures | Lines 163–165: survey-weighted MetS prevalence (men 42.5%; women 57.1%; p<0.001) and unweighted values reported in Table 1 (40.6% and 53.5%). Low muscle mass prevalences: lines 174–182 and Fig 1 (caption at lines 183–188). |
| Main results | 16 | (*a*) Give unadjusted estimates and, if applicable, confounder-adjusted estimates and their precision (eg, 95% confidence interval). Make clear which confounders were adjusted for and why they were included | Lines 216–237 (logistic regression for MetS, Tables 4 and 5 placed in read order); covariates listed in Methods (lines 148–155); 95% CIs reported throughout. Adjustment for age, smoking, alcohol, and physical activity prespecified based on established lifestyle determinants of metabolic risk. |
|  |  | (*b*) Report category boundaries when continuous variables were categorized | Lines 117–121 (Class I/II low muscle mass cut-offs explicitly stated for ASMI and ALM/TLM, both sexes); lines 131–134 (FMI category thresholds). |
|  |  | (*c*) If relevant, consider translating estimates of relative risk into absolute risk for a meaningful time period | n/a – cross-sectional design; survey-weighted prevalences are reported (Figs 2–3), but absolute risk over a time period is not calculated. |
| Other analyses | 17 | Report other analyses done—eg analyses of subgroups and interactions, and sensitivity analyses | Lines 238–254 and Figs 2–3 (BMI- and FMI-stratified linear trend tests); lines 273–281 (physical activity-stratified analyses with formal interaction tests; men p=0.231, women p=0.945); supplementary analyses cited at lines 232–254 (S4–S6, S8 Tables in S1 File). |
| Discussion | | | |
| Key results | 18 | Summarise key results with reference to study objectives | Lines 282–290: Discussion paragraph 1 (key results aligned with the prespecified objective on the impact of normalization strategy). |
| Limitations | 19 | Discuss limitations of the study, taking into account sources of potential bias or imprecision. Discuss both direction and magnitude of any potential bias | Lines 346–371: cross-sectional design; lack of functional measures for sarcopenia diagnosis; selection bias from DXA participation (with quantitative comparison to general-population MetS estimates); missing medication data; single HOMA-IR; self-reported physical activity; absence of MRI/CT validation of TLM stability. |
| Interpretation | 20 | Give a cautious overall interpretation of results considering objectives, limitations, multiplicity of analyses, results from similar studies, and other relevant evidence | Lines 291–345: Discussion paragraphs 2–5 (mechanistic interpretation; supporting evidence from supplementary analyses; links to ESPEN-EASO sarcopenic-obesity consensus and prior counterintuitive NHANES findings); ALM/TLM positioned as a candidate adiposity-resistant index, not a clinically validated sarcopenia threshold. |
| Generalisability | 21 | Discuss the generalisability (external validity) of the study results | Lines 355–371: limitations note higher MetS prevalence in DXA subsample compared with general-population estimates; need for external replication and prospective validation. Conclusions (lines 376–385): findings to be “confirmed in external cohorts and prospective studies.” |
| Other information | | | |
| Funding | 22 | Give the source of funding and the role of the funders for the present study and, if applicable, for the original study on which the present article is based | Funding information is provided through the PLOS ONE submission system per journal policy. The study was unfunded: “The authors received no specific funding for this work.” |

*Give information separately for exposed and unexposed groups.

**Note:** An Explanation and Elaboration article discusses each checklist item and gives methodological background and published examples of transparent reporting. The STROBE checklist is best used in conjunction with this article (freely available on the Web sites of PLoS Medicine at http://www.plosmedicine.org/, Annals of Internal Medicine at http://www.annals.org/, and Epidemiology at http://www.epidem.com/). Information on the STROBE Initiative is available at www.strobe-statement.org.
