# Supplementary material for: Appendicular-to-trunk lean mass ratio resolves the height-based normalization paradox in low muscle mass and metabolic syndrome: A population-based cross-sectional study
Source: PLoS One. 2026 Aug 25;21(8):e0357017. doi: 10.1371/journal.pone.0357017 (PMC13505910; doi:10.1371/journal.pone.0357017)
Supplement: Peer review history

# Peer review: Decision Letter 0

**DOI:** 10.1371/journal.pone.0357017.r001

**Authors:** Girish C Melkani

21 Jul 2026

Dear Dr. Hong,

Thank you for submitting your manuscript to PLOS ONE. After careful consideration, we feel that it has merit but does not fully meet PLOS ONE’s publication criteria as it currently stands. Therefore, we invite you to submit a revised version of the manuscript that addresses the points raised during the review process.

Please submit your revised manuscript by Aug 20 2026 11:59PM. If you will need more time than this to complete your revisions, please reply to this message or contact the journal office at plosone@plos.org. When you're ready to submit your revision, log on to https://www.editorialmanager.com/pone/ and select the 'Submissions Needing Revision' folder to locate your manuscript file.

- A letter that responds to each point raised by the academic editor and reviewer(s). You should upload this letter as a separate file labeled 'Response to Reviewers'.
- A marked-up copy of your manuscript that highlights changes made to the original version. You should upload this as a separate file labeled 'Revised Manuscript with Track Changes'.
- An unmarked version of your revised paper without tracked changes. You should upload this as a separate file labeled 'Manuscript'.

If you would like to make changes to your financial disclosure, please include your updated statement in your cover letter. Guidelines for resubmitting your figure files are available below the reviewer comments at the end of this letter.

If applicable, we recommend that you deposit your laboratory protocols in protocols.io to enhance the reproducibility of your results. Protocols.io assigns your protocol its own identifier (DOI) so that it can be cited independently in the future. For instructions see: https://journals.plos.org/plosone/s/submission-guidelines#loc-laboratory-protocols. Additionally, PLOS ONE offers an option for publishing peer-reviewed Lab Protocol articles, which describe protocols hosted on protocols.io. Read more information on sharing protocols at https://plos.org/protocols?utm_medium=editorial-email&utm_source=authorletters&utm_campaign=protocols.

As the corresponding author, your ORCID iD is verified in the submission system and will appear in the published article. PLOS supports the use of ORCID, and we encourage all coauthors to register for an ORCID iD and use it as well. Please encourage your coauthors to verify their ORCID iD within the submission system before final acceptance, as unverified ORCID iDs will not appear in the published article. *Only*  the individual author can complete the verification step; PLOS staff *cannot*  verify ORCID iDs on behalf of authors.

We look forward to receiving your revised manuscript.

Kind regards,

Girish C Melkani, PhD

Academic Editor

PLOS One

Journal Requirements:

When submitting your revision, we need you to address these additional requirements.

1. Please ensure that your manuscript meets PLOS ONE's style requirements, including those for file naming. The PLOS ONE style templates can be found at

https://journals.plos.org/plosone/s/file?id=wjVg/PLOSOne_formatting_sample_main_body.pdf and https://journals.plos.org/plosone/s/file?id=ba62/PLOSOne_formatting_sample_title_authors_affiliations.pdf

2. You have indicated that data is available from [knhanes@kdca.go.kr]. Please can we ask you to provide us with a general contact email address for the data requests, so readers can request access in perpetuity. If a general email is not available please provide a link to a website where readers can obtain access to data.

3. Thank you for stating the following in your Competing Interests section:

None

Please complete your Competing Interests on the online submission form to state any Competing Interests. If you have no competing interests, please state "The authors have declared that no competing interests exist.", as detailed online in our guide for authors at http://journals.plos.org/plosone/s/submit-now

This information should be included in your cover letter; we will change the online submission form on your behalf.

4. Thank you for stating the following in the Acknowledgments Section of your manuscript:

The authors thank the Korea Disease Control and Prevention Agency for providing access to the KNHANES 2008–2010 data.

We note that you have provided funding information that is not currently declared in your Funding Statement. However, funding information should not appear in the Acknowledgments section or other areas of your manuscript. We will only publish funding information present in the Funding Statement section of the online submission form.

Please remove any funding-related text from the manuscript and let us know how you would like to update your Funding Statement. Currently, your Funding Statement reads as follows:

The author(s) received no specific funding for this work.

Please include your amended statements within your cover letter; we will change the online submission form on your behalf.

5. If the reviewer comments include a recommendation to cite specific previously published works, please review and evaluate these publications to determine whether they are relevant and should be cited. There is no requirement to cite these works unless the editor has indicated otherwise.

6. Please review your reference list to ensure that it is complete and correct. If you have cited papers that have been retracted, please include the rationale for doing so in the manuscript text, or remove these references and replace them with relevant current references. Any changes to the reference list should be mentioned in the rebuttal letter that accompanies your revised manuscript. If you need to cite a retracted article, indicate the article’s retracted status in the References list and also include a citation and full reference for the retraction notice.

Additional Editor Comments :

Dear Authors,

Your manuscript has been reviewed again by additional reviewers and previous reviewers were not available. Based on the reviewers feedback, addressed point-by-point response raised by each reviewer and make changes in the manuscript. All the best.

[Note: HTML markup is below. Please do not edit.]

Reviewers' comments:

Reviewer's Responses to Questions

**Comments to the Author**

1. Is the manuscript technically sound, and do the data support the conclusions?

Reviewer #1: No

Reviewer #2: Yes

**********

2. Has the statistical analysis been performed appropriately and rigorously?

Reviewer #1: I Don't Know

Reviewer #2: Yes

**********

3. Have the authors made all data underlying the findings in their manuscript fully available?

The PLOS Data policy

Reviewer #1: No

Reviewer #2: Yes

**********

4. Is the manuscript presented in an intelligible fashion and written in standard English?

Reviewer #1: No

Reviewer #2: Yes

**********

Reviewer #1: Comments:

The author has presented thoughtful ideas, and the work is appreciated for its interpretation and results. However, there are still several areas that require improvement. The manuscript would benefit from further refinement in clarity, structure, and depth of analysis to strengthen its overall quality and impact.

Abstract: (line 29-33) ASMI validity concern: ASMI shows strong correlations with adiposity (BMI/FMI) and paradoxical protective associations with metabolic syndrome, suggesting it may not represent muscle mass but a body-size/adiposity proxy. Interpretation should be substantially revised.

Introduction: (line 48-50) Please Include recent high-quality journal references in the Introduction to strengthen background and support study rationale

Study Designed: The study population should be clearly divided into mutually exclusive categories (non-users, single exposure (like only alcohol or smokers), and dual exposure groups alcohol +smokers and heavy or small quantity) with sample sizes and percentages.

Results: Do you have data on participants' dietary habits? If yes, could dietary habits (fried/oil-based versus non-fried food consumption) be systematically classified and reported alongside smoking and alcohol status?

Figures: Please include a Venn diagram to clearly show overlap between exposure groups (smoking, alcohol, and dietary habits) with proper population stratification.

Reviewer #2: This manuscript addresses an important question of how to normalize appendicular lean mass so that is more reflective of true metabolic health. The study is carefully conducted, the statistical analyses are generally appropriate, and the central findings are clearly presented. However, there are some lapses.

Incorporation of TLM in body mass normalization is the main selling point of this paper. However, TLM might not be as stable of a denominator because it itself can change with the effects of aging, disease and obesity on visceral organs and trunk muscle. As such, the utility of the proposed index should be discussed more carefully. While the authors acknowledge this limitation, it is central to the interpretation of ALM/TLM and deserves greater emphasis. ASMI still remains widely used in major sarcopenia frameworks and has been associated with important functional outcomes in numerous studies. Readers may benefit from a clearer discussion that distinguishes metabolic validity from functional validity. An index that performs poorly for metabolic risk delineation may still perform adequately for identifying weakness, mobility impairment, or other sarcopenia-related outcomes. This distinction would provide a more balanced perspective regarding the strengths and weaknesses of different normalization approaches.

The manuscript demonstrates that ALM/TLM is associated with metabolic syndrome and HOMA-IR, but it remains unknown whether ALM/TLM predicts clinically meaningful outcomes better than ASMI that curtails it from being a clinical adoption recommendation.

The external validity deserves additional consideration. The study size is large but the population consists exclusively of older Korean adults. Because body composition, adiposity distribution, and sarcopenia prevalence vary substantially across ethnic groups, the generalizability of the proposed ALM/TLM cutoffs to other populations remains uncertain. A brief discussion of this limitation would strengthen the manuscript.

**********

what does this mean?). If published, this will include your full peer review and any attached files.

If you choose “no”, your identity will remain anonymous but your review may still be made public.

**Do you want your identity to be public for this peer review?**  For information about this choice, including consent withdrawal, please see our Privacy Policy

Reviewer #1: No

Reviewer #2: **Yes:**  Sachin Budhathoki

**********

[NOTE: If reviewer comments were submitted as an attachment file, they will be attached to this email and accessible via the submission site. Please log into your account, locate the manuscript record, and check for the action link "View Attachments". If this link does not appear, there are no attachment files.]

To ensure your figures meet our technical requirements, please review our figure guidelines: https://journals.plos.org/plosone/s/figures

You may also use PLOS’s free figure tool, NAAS, to help you prepare publication quality figures: https://journals.plos.org/plosone/s/figures#loc-tools-for-figure-preparation.

NAAS will assess whether your figures meet our technical requirements by comparing each figure against our figure specifications.

Attachment Submitted filename: Article comments.pdf

# Author comment: Author response to Decision Letter 1

**DOI:** 10.1371/journal.pone.0357017.r002

3 Aug 2026

Response to Reviewers

Manuscript ID: PONE-D-26-25084

Title: Appendicular-to-trunk lean mass ratio resolves the height-based normalization paradox in low muscle mass and metabolic syndrome: A population-based cross-sectional study

Corresponding author: Sangmo Hong, MD, PhD

Dear Editors,

We thank the Academic Editor and both reviewers for their careful and constructive evaluation of our manuscript. As we agree with the comments raised, we have revised the manuscript accordingly. Below we respond to each point in a point-by-point manner. Reviewer and editor comments are shown in italics; revised or newly added manuscript text is shown as From/To pairs, with the key changed portions highlighted in yellow. Newly added references are listed at the end.

Journal Requirements

Requirement 1: Please ensure that your manuscript meets PLOS ONE’s style requirements, including those for file naming.

Response: We have reformatted the manuscript, tables, and supporting information to conform to the PLOS ONE style templates and have renamed all files according to PLOS ONE conventions (Manuscript, Response to Reviewers, Revised Manuscript with Track Changes, S1 File, S1 Checklist, and figure files).

Requirement 2: Please provide a general contact address or a website where readers can obtain access to the data in perpetuity.

Response: We have revised the Data Availability Statement to direct readers to the public KNHANES portal rather than a personal email address. The revised statement reads: “The data underlying this study are third-party data owned by the Korea Disease Control and Prevention Agency and are publicly available to all researchers from the Korea National Health and Nutrition Examination Survey (KNHANES) website (https://knhanes.kdca.go.kr; English: https://knhanes.kdca.go.kr/knhanes/eng/) following free registration. The authors had no special access privileges.”

Requirement 3: If you have no competing interests, please state the required sentence.

Response: The authors have no competing interests. We confirm the statement: “The authors have declared that no competing interests exist.”

Requirement 4: Funding information should not appear in the Acknowledgments. Please confirm your Funding Statement.

Response: This study received no specific funding. The Funding Statement remains: “The authors received no specific funding for this work.” The Acknowledgments sentence thanking the KDCA refers solely to provision of, and access to, the publicly available KNHANES data and does not describe financial support; we have clarified this wording to avoid ambiguity.

Requirement 5: If reviewer comments recommend citing specific works, evaluate their relevance and cite if appropriate.

Response: We evaluated each suggested area and added recent, relevant, high-quality references to the Introduction and Discussion (listed at the end of this letter). Each was assessed for direct relevance before inclusion.

Requirement 6: Please review your reference list to ensure it is complete and correct, and check for retracted articles.

Response: We re-checked all references for completeness and accuracy and confirmed none has been retracted. We also corrected the citation numbering so references appear strictly in order of first appearance (this had affected the ordering of the sarcopenia-consensus and ESPEN–EASO references), and integrated the newly added references into the sequence.

Reviewer #1

We thank Reviewer #1 for recognizing the interpretation and results of our work and for the suggestions to improve clarity, structure, and depth of analysis.

Comment 1: ASMI shows strong correlations with adiposity (BMI/FMI) and paradoxical protective associations with metabolic syndrome, suggesting it may not represent muscle mass but a body-size/adiposity proxy. Interpretation should be substantially revised.

Response: Thank you for this important comment. We agree that the original wording required a more explicit and cautious interpretation. We revised the concluding portion of the Abstract to clarify that the inverse association between ASMI-defined low muscle mass and MetS may reflect the adiposity-dependent properties of height-based normalization and should not be interpreted as metabolic protection. We also clarified that our findings concern the metabolic behavior of ASMI and do not negate its established role in functional sarcopenia assessment. The revised Abstract remains within the 300-word limit.

From: Height-based normalization produced a paradoxical inverse association between low muscle mass and MetS, whereas ALM/TLM yielded directionally consistent positive associations across adiposity strata. These findings indicate that the choice of normalization strategy substantially affects the apparent metabolic significance of low muscle mass and support ALM/TLM as a candidate adiposity-resistant index for further evaluation.

To: ASMI correlated positively with adiposity, while ASMI-defined low muscle mass was inversely associated with MetS, suggesting that ASMI may partly reflect body size/adiposity rather than relative muscle depletion alone; this inverse association should not be interpreted as protective. By contrast, ALM/TLM-defined low muscle mass showed consistently positive associations with MetS across adiposity strata, supporting further evaluation of ALM/TLM, although its clinical validity remains unestablished.

Additional correction (same Abstract sentence): the men ASMI Class I odds ratio was harmonized with Table 4/S4.

From: …men odds ratio [OR] 0.500, 95% CI 0.391–0.639…

To: …men odds ratio [OR] 0.496, 95% CI 0.388–0.636…

Comment 2: Please include recent high-quality journal references in the Introduction to strengthen background and support study rationale.

Response: Thank you for this helpful suggestion. We revised this passage for greater precision and added recent references matched to specific claims, replacing “not metabolically neutral” with the more precise “not independent of adiposity.”

From: This question is difficult to address directly because commonly used lean-mass indices are not metabolically neutral. Their values are influenced, to varying degrees, by fat mass itself. As a result, the apparent metabolic significance of low muscle mass may differ substantially depending on how appendicular lean mass (ALM) is normalized, and the independent contribution of muscle depletion may be obscured by the adiposity dependence of the index used.

To: This question is difficult to address directly because commonly used lean-mass indices are not independent of adiposity. Greater abdominal adiposity may coexist with higher ALM but poorer muscle quality [3], and fat mass and fat-free mass tend to increase concurrently and should be interpreted in relation to one another [4]. Consequently, the apparent metabolic significance of low muscle mass may differ substantially depending on how ALM is normalized, making the muscle-specific contribution to metabolic risk difficult to isolate when the index used remains dependent on adiposity.

Comment 3: The study population should be clearly divided into mutually exclusive categories (non-users, single exposure, and dual exposure) with sample sizes and percentages.

Response: Thank you for this helpful comment. We added S9 Table, which cross-classifies participants into four mutually exclusive categories based on current smoking and alcohol consumption ≥2 times/week, with sex-specific sample sizes and overall percentages. We also examined whether the association between ALM/TLM class and metabolic syndrome (MetS) varied across these categories where the interaction was estimable. In main-effects models, ALM/TLM-defined low muscle mass remained associated with MetS in both sexes (Classes I and II versus normal: men, OR 1.95 [95% CI 1.44–2.65] and 2.43 [1.76–3.36]; women, OR 1.51 [1.16–1.98] and 2.80 [1.79–4.40]; Wald F for ALM/TLM class, P<0.001 in both sexes). The interaction did not reach statistical significance in men (P=0.054). In women, it was not estimable because the model did not converge owing to sparse cells, including only eight women with both exposures. These main-effects estimates use the four-category lifestyle variable in place of the separate smoking and alcohol covariates in Table 4 and should be interpreted as results from a distinct lifestyle-adjusted model.

Added to Methods: In a supplementary analysis, participants were cross-classified into four mutually exclusive categories based on current smoking and alcohol consumption ≥2 times/week; this joint variable was used both as an adjustment covariate and, where estimable, to assess effect modification of the ALM/TLM–MetS association (sex-stratified complex-samples logistic regression; S9 Table).

Added to Results: The mutually exclusive smoking–alcohol distribution is presented by sex in S9 Table. Adjustment using this joint lifestyle variable did not change the direction or statistical significance of the association between ALM/TLM-defined low muscle mass and MetS in either sex; the interaction was not statistically significant in men and could not be reliably estimated in women.

Comment 4: Do you have data on participants’ dietary habits? Could dietary habits (fried/oil-based versus non-fried food) be systematically classified and reported?

Response: Thank you for this comment. KNHANES 2008–2010 does not provide a validated classification of fried or oil-based food consumption. We therefore conducted a sensitivity analysis using the percentage of energy derived from fat based on the 24-hour dietary recall. Additional adjustment for this variable did not materially alter the association between ALM/TLM-defined low muscle mass and MetS in either sex, and the dietary-fat variable was not itself associated with MetS (men, P=0.445; women, P=0.627; S10 Table). We have added this analysis to the Methods and Results.

Added to Methods: In a complete-case dietary subsample, we examined whether additional adjustment for the percentage of energy derived from fat changed the ALM/TLM–MetS estimates (S10 Table).

Added to Results: Additional adjustment for the percentage of energy derived from fat did not materially alter the ALM/TLM–MetS association in either sex (S10 Table).

Comment 5: Please include a Venn diagram to clearly show overlap between exposure groups (smoking, alcohol, and dietary habits) with proper population stratification.

Response: Thank you for this suggestion. Because the available dietary-fat measure is continuous and no validated fried-food category was available, it could not be represented as a discrete set without arbitrary categorization. We therefore added a sex-stratified supplementary diagram showing the overlap between current smoking and alcohol consumption ≥2 times/week (S1 Fig), while dietary fat intake is addressed separately in S10 Table.

S1 Fig legend: Overlap between current smoking and alcohol consumption ≥2 times/week, by sex. (a) Men. (b) Women. Numbers indicate participants in each mutually exclusive category. Participants with complete smoking and alcohol data: men, n=1,372; women, n=1,802.

Reviewer #2

We are grateful to Reviewer #2 for the positive assessment and for comments that sharpen the interpretation of the study.

Comment 1: TLM might not be as stable a denominator because it can change with aging, disease, and obesity on visceral organs and trunk muscle. While acknowledged, this limitation is central to the interpretation of ALM/TLM and deserves greater emphasis.

Response: Thank you for emphasizing this important point. We agree that uncertainty regarding the TLM denominator is central to the interpretation of ALM/TLM and have given it greater prominence in the Discussion. We now clarify that TLM cannot be regarded as a fixed or biologically inert denominator because lean-mass measures vary with age and body size, DXA-derived trunk lean mass may be influenced by hydration, and central lean-mass distribution has itself been associated with adverse outcomes. We also clarify that a low ALM/TLM value may reflect lower appendicular lean mass, higher trunk lean mass, or both. Accordingly, we present ALM/TLM as a candidate normalizer requiring component-level imaging and longitudinal validation.

From: The observed negative correlation between ALM/TLM and FMI (men r=−0.199; women r=−0.243) is consistent with this interpretation. However, it does not constitute direct proof, and imaging-based validation of TLM stability across adiposity strata is needed before this rationale can be confirmed.

To: The modest inverse correlations between ALM/TLM and FMI (men, r=−0.199; women, r=−0.243) indicate that ALM/TLM does not track adiposity in the same direction as ASMI; however, they do not establish independence from adiposity. Importantly, TLM should not be regarded as a fixed or biologically inert denominator. Central lean-mass distribution has itself been associated with adverse outcomes [19], indicating that the denominator may carry clinically relevant information; moreover, lean-mass measures vary with age and body size [20], and DXA-derived trunk lean mass may be influenced by hydration [21]. Consequently, a low ALM/TLM value may reflect lower ALM, higher TLM, or both. Component-level imaging and longitudinal validation are therefore required before ALM/TLM can be regarded as a stable or muscle-specific normalization index.

Comment 2: ASMI remains widely used and is associated with functional outcomes. A clearer distinction between metabolic validity and functional validity would provide a more balanced perspective.

Response: Thank you for this constructive suggestion. We have added a paragraph distinguishing the metabolic associations examined in our study from the functional and clinical roles of muscle-mass indices. We now clarify that ASMI remains the muscle-mass criterion in major sarcopenia frameworks, in which muscle strength and physical performance are assessed separately. Our findings therefore do not diminish the established role of ASMI within these frameworks; rather, they suggest that ALM/TLM may provide complementary information regarding metabolic-risk associations but requires validation against functional outcomes.

Added to Discussion: Our findings concern the metabolic associations of these indices and should be distinguished from their functional and clinical roles. ASMI remains the muscle-mass criterion in major sarcopenia frameworks [24, 25], in which muscle strength and physical performance are assessed separately. An index showing limited or counterintuitive associations with metabolic risk in a given setting may still retain value as a measure of muscle quantity within a multidimensional sarcopenia assessment. Our findings therefore do not diminish the established role of ASMI within current frameworks. Rather, they suggest that ALM/TLM may provide complementary information regarding metabolic-risk associations and should be evaluated alongside, rather than as a replacement for, height-based indices.

Comment 3: It remains unknown whether ALM/TLM predicts clinically meaningful outcomes better than ASMI, which curtails it from being a clinical adoption recommendation.

Response: Thank you for this important point. We agree that it remains unknown whether ALM/TLM predicts clinically meaningful outcomes, or does so better than ASMI, precluding any recommendation for clinical use. We have made this explicit in the Conclusions and now state that ALM/TLM requires external, prospective, outcome-based validation. For consistency, we also revised a sentence in the Discussion that had referred to potential clinical settings, so that ALM/TLM is now framed for research evaluation only. This clarification complements the existing limitation that the proposed categories are not clinically validated sarcopenia thresholds.

From: …and support ALM/TLM as a candidate adiposity-resistant index for further evaluation in research and clinical settings concerned with sarcopenic obesity. Given the cross-sectional design and the absence of functional measures required for sarcopenia diagnosis, the findings should be interpreted cautiously and confirmed in external cohorts and prospective studies.

To: These findings indicate that normalization strategies substantially affect the apparent me

# Editor report: Decision Letter 1

**DOI:** 10.1371/journal.pone.0357017.r003

**Authors:** Girish C Melkani

12 Aug 2026

Appendicular-to-trunk lean mass ratio resolves the height-based normalization paradox in low muscle mass and metabolic syndrome: A population-based cross-sectional study

PONE-D-26-25084R1

Dear Dr. Hong,

We’re pleased to inform you that your manuscript has been judged scientifically suitable for publication and will be formally accepted for publication once it meets all outstanding technical requirements.

Within one week, you’ll receive an e-mail detailing the required amendments. When these have been addressed, you’ll receive a formal acceptance letter and your manuscript will be scheduled for publication.

An invoice will be generated when your article is formally accepted. Please note, if your institution has a publishing partnership with PLOS and your article meets the relevant criteria, all or part of your publication costs will be covered. Please make sure your user information is up-to-date by logging into Editorial Manager at Editorial Manager® and clicking the ‘Update My Information' link at the top of the page. For questions related to billing, please contact billing support.

If your institution or institutions have a press office, please notify them about your upcoming paper to help maximize its impact. If they’ll be preparing press materials, please inform our press team as soon as possible -- no later than 48 hours after receiving the formal acceptance. Your manuscript will remain under strict press embargo until 2 pm Eastern Time on the date of publication. For more information, please contact onepress@plos.org.

Kind regards,

Girish C Melkani, PhD

Academic Editor

PLOS One

# Editor report: Acceptance letter

**DOI:** 10.1371/journal.pone.0357017.r004

**Authors:** Girish C Melkani

PONE-D-26-25084R1

PLOS One

Dear Dr. Hong,

I'm pleased to inform you that your manuscript has been deemed suitable for publication in PLOS One. Congratulations! Your manuscript is now being handed over to our production team.

At this stage, our production department will prepare your paper for publication. This includes ensuring the following:

* All references, tables, and figures are properly cited

* All relevant supporting information is included in the manuscript submission,

* There are no issues that prevent the paper from being properly typeset

You will receive further instructions from the production team, including instructions on how to review your proof when it is ready. Please keep in mind that we are working through a large volume of accepted articles, so please give us a few days to review your paper and let you know the next and final steps.

Lastly, if your institution or institutions have a press office, please let them know about your upcoming paper now to help maximize its impact. If they'll be preparing press materials, please inform our press team within the next 48 hours. Your manuscript will remain under strict press embargo until 2 pm Eastern Time on the date of publication. For more information, please contact onepress@plos.org.

You will receive an invoice from PLOS for your publication fee after your manuscript has reached the completed accept phase. If you receive an email requesting payment before acceptance or for any other service, this may be a phishing scheme. Learn how to identify phishing emails and protect your accounts at https://explore.plos.org/phishing.

If we can help with anything else, please email us at customercare@plos.org.

Thank you for submitting your work to PLOS One and supporting open access.

Kind regards,

PLOS ONE Editorial Office Staff

on behalf of

Dr. Girish C Melkani

Academic Editor

PLOS One
